# Exposure States, Environmental Fate, and Functional Effects of Microplastics in Soil: Bibliometric Mapping and Critical Mechanistic Synthesis

**DOI:** 10.3390/polym18192364

**Published:** 2026-09-28

**Authors:** Yuhe Li, Su Yan, Xinting Lv, Yuxin Tian, Mingji Jin, Yue Quan, Yiran Zheng

**Affiliations:** 1 Department of Geography and Ocean Sciences, Yanbian University, Hunchun 133300, China; liyuhe1102@163.com (Y.L.); jinmingji@ybu.edu.cn (M.J.); 2 Department of Resource Utilization and Plant Protection, Yanbian University, Yanji 133002, China; 15843615021@163.com (S.Y.); 15545240805@163.com (X.L.); 18744351618@163.com (Y.T.)

**Keywords:** microplastics, soil, exposure state, environmental fate

## Abstract

Reported effects of microplastics in soil vary with particle properties, exposure conditions, and measured endpoints. We combined bibliometric mapping with a critical mechanistic synthesis to examine these differences. A 3200-record Web of Science Core Collection corpus was tested against broad (4787) and title-strict (2375) sensitivity corpora. A year-stratified manual relevance audit judged 115 of 120 sampled records (95.8%) to be in scope. An article-only subset of 2756 records retained positive directions for all twelve principal title-concept trends and preserved 112 of 119 primary co-citation nodes. Twelve title concepts increased consistently across corpus definitions and start years, led by plant and crop performance, biodegradable plastics, and aging and weathering. Only co-contamination and nitrogen cycling remained significant under every robust model specification. The mechanistic synthesis indicates that differences in particle state, soil-mediated contact, endpoints, and inferential scope can contribute to apparently contradictory findings. We propose an exposure-to-function framework that integrates exposure descriptors with a bounded sequence linking particle characterization, fate and biological contact, process change, functional consequences, and persistence or recovery. Risk and management claims should remain within the links supported by evidence.

## 1. Introduction

Microplastics are plastic particles commonly defined as smaller than 5 mm and occurring as fragments, fibers, films, foams, or beads. The term denotes a size-defined class; polymer identity and particle morphology are separate descriptors that influence environmental behavior. Polyethylene, polypropylene, polystyrene, polyethylene terephthalate, polyvinyl chloride, and biodegradable polymers can all occur as microplastics, but their behavior in soil differs. Polymer chemistry and additives influence density, flexibility, degradation, and sorption, while particle size, shape, and aging influence surface area, pore entry, fragmentation, and biological contact [1,2,3].

Field observations show that microplastics occur within soil aggregates [4]. Agricultural inputs include residues from plastic mulch and other agricultural products, whereas sewage sludge and other waste-derived amendments provide additional pathways [5,6]. Irrigation, atmospheric deposition, road dust, litter fragmentation, landfill leakage, runoff, flooding, and wind erosion are additional pathways described in field and review evidence [6,7,8]. Mechanically generated particles used in controlled experiments represent a separate material history rather than a field source [9]. These inputs deliver different mixtures of polymers, fibers, films, and fragments; a bulk concentration alone therefore cannot specify particle composition or exposure history. Continued plastic production and waste generation sustain these inputs at regional and global scales [10].

After entering soil, microplastics may be retained in surface horizons, incorporated into aggregates, moved through preferential flow, redistributed by tillage and soil fauna, or transported by runoff and wind [6,8,11]. Aging, mineral coatings, biofilms, and contact with natural organic matter change particle surfaces and can alter retention, fragmentation, and contaminant sorption [12]. Movement through pore networks is further regulated by soil structure and hydrological conditions [13,14,15]. Fate is therefore controlled jointly by particle properties and soil texture, aggregation, water regime, pH, ionic strength, organic matter, vegetation, and management.

Under specific experimental conditions, soil microplastics have been associated with changes in bulk density, aggregation, pore connectivity, water retention, hydraulic conductivity, and aeration [16,17,18], as well as dissolved organic matter, nutrient availability, enzyme activity, and microbial habitat [19,20,21]. These changes may influence carbon and nitrogen cycling, but their direction and magnitude vary with polymer type, particle form, dose, soil conditions, and exposure duration [22,23,24].

Plants, microorganisms, and soil fauna respond to both particles and the soil conditions modified by them. Changes in germination, root architecture, nutrient acquisition, and growth have been reported under specific treatments [25,26,27]. Bacterial-community and enzyme responses have been observed for defined particle–soil combinations [20], and soil fauna can ingest or redistribute particles [28,29]. Recent studies of biodegradable microplastics, nanoplastics, and plastic additives further show that particle type and exposure route can alter rhizosphere microbiomes, metabolite profiles, and plant performance [30,31]. Responses can extend from rhizosphere processes to whole-plant performance [32]. Because lower size boundaries vary among definitions, nanoplastic studies are used here only when they clarify mechanisms relevant to microplastic transformation or contact and are not treated as direct evidence for all microplastic size classes.

Existing reviews have cataloged the sources, occurrence, analytical methods, and potential effects of soil microplastics [33,34,35], while other syntheses have identified ecological implications and knowledge gaps [36,37]. Several methodological reviews already recommend reporting individual descriptors such as polymer identity, particle size and shape, analytical recovery, soil type, and exposure duration [33,34,35]. The unresolved problem is therefore not the absence of these descriptors individually, but their integration to provide a consistent basis for cross-study comparison and for limiting claims to what was actually measured. This review asks which combinations of particle characteristics, soil conditions, and observation designs are associated with different functional responses and how far the resulting conclusions can legitimately extend along an evidence chain.

This review uses exposure state as an operational unit of comparison. It comprises five descriptor groups: material identity (polymer and additives), particle attributes (size, shape, and surface state), exposure history (source, applied amount, aging, and prior transport), soil context (physical, chemical, hydrological, and biological conditions), and spatiotemporal observation context (sampling depth, exposure duration, and timing of measurement). These descriptors specify the conditions needed to connect a measured particle assemblage to a soil process or biological outcome; they do not form a numerical risk score. Persistence or recovery is evaluated downstream as an outcome. The contribution is not that each descriptor is new, but that the descriptors are integrated with a bounded sequence from source-defined particle state → transformation and redistribution in soil → biological and process contact → functional response → persistence or recovery → risk or management inference.

This review first maps the development of soil-microplastic research using a screened Web of Science Core Collection (WoSCC) corpus and scope-sensitivity analyses. It then critically synthesizes experimental and field evidence to examine how particle characteristics, soil-mediated fate and contact, and observation design influence reported functional responses. The bibliometric and mechanistic components answer complementary questions; title-concept occurrence frequency is not used as evidence of environmental effect. Records indexed in the incomplete 2026 period remain within the continuous corpus but are used only for descriptive signals.

## 2. Review Design and Methods

### 2.1. Database and Search Strategy

The Web of Science Core Collection (WoSCC) was searched with TS = ((microplastics OR microplastic) AND (soils OR soil OR ground)). The search was frozen on 3 September 2026, spanned 2013–2026, and applied no language or document-type restrictions. Twelve non-overlapping export batches were saved as full records with cited references. They contained 5629 unique WoS accession numbers (UTs); no duplicate UT occurred across the canonical batches. One online-first record assigned to 2027 was retained in the raw archive but excluded from the declared period, leaving 5628 temporally eligible records. The broad-term ground was retained in the prespecified, recall-oriented query but was not assumed to be soil-specific. This study is a critical bibliometric–mechanistic review, not a PRISMA systematic review or effect-size meta-analysis.

### 2.2. Data Processing and Bibliometric Analysis

#### 2.2.1. Corpus Assembly and Annual Analysis

Scope precision was addressed with deterministic, record-level rules rather than by treating all 5628 retrieved records as equivalent evidence. The primary corpus retained a record when (i) both microplastic and soil/agricultural anchors occurred in the title; (ii) a soil-anchored title was supported by at least two microplastic mentions in the abstract or author keywords; or (iii) a microplastic-anchored title was supported by strong soil evidence in the title/abstract/author-keyword fields and by either a terrestrial title proxy or at least two strong soil mentions in the abstract or author keywords. Records marked as retracted or carrying an expression of concern were excluded, as were titles dominated by a prespecified non-soil lexicon unless a strong soil title anchor was present. The rules and lexicons are supplied in code and Supplementary Tables S1–S3. They produced 3200 primary records; a broader title/abstract/author-keyword corpus (4787) and a title-strict corpus (2375) served as alternative sensitivity bounds. The primary corpus contained 2738 records indexed as Article, 434 as Review, 18 as Article; Early Access, and 10 as Review; Early Access. Of the 3200 records, 3196 were in English. Field coverage was 3200/3200 for titles, years, document types, languages, and accession numbers; 3197/3200 for abstracts; 3199/3200 for cited references; and 3046/3200 (95.2%) for author keywords.

To provide an independent human check of corpus relevance without re-screening the full database, a 120-record sample was drawn from the frozen 3200-record primary corpus with representation across publication years. One author reviewed the title and abstract of each sampled record and answered a single prespecified question: whether the record belonged to the soil-microplastic research field. Records explicitly flagged as ambiguous or partially off-scope were conservatively coded as not in scope. The audit was conducted after the corpus and analysis rules had been frozen and was not used to optimize the observed title-concept occurrence trends or redefine the primary corpus. Because the audit sampled retained records only and used one evaluator, it is interpreted as a relevance check rather than an estimate of recall or inter-reviewer agreement (Supplementary Table S11).

#### 2.2.2. Co-Citation, Keyword, and Collaboration Networks

The original analyses were performed in Python 3.13.9 and NetworkX 3.6.1 from the same 3200-record primary corpus. NumPy 2.3.4 and pandas 2.3.3 supported numerical and tabular processing; Matplotlib 3.10.7, seaborn 0.13.2 and Pillow 12.0.0 supported plotting and image export. The author-keyword network used only WoS author keywords (DE), because Keywords Plus can import peripheral indexing terms. Only the exact standalone search anchors microplastic(s) and soil(s) were removed from display; observed composite phrases such as microplastic pollution were retained. Keywords occurring in at least eight records were eligible, co-occurrence required at least three records, and association strength was *n* × *c_ij_*/(*f_i_* × *f_j_*), where *n* is the number of records, *c_ij_* is the number containing both terms, and *f_i_* and *f_j_* are their record frequencies; the inclusion threshold was 0.8. The largest connected component contained 107 nodes and 216 analysis edges. Louvain detection used resolution 1.0; the primary seed was 20,260,903, and robustness used seeds 20,260,903 + 97*k* (*k* = 0,…,9). A top-five-per-node plus maximum-spanning-forest backbone (167 edges) was used only for visualization. For the author-keyword visualization only, an ordered, versioned dictionary assigned each eligible keyword to the first matching one of six named display domains; unmatched keywords were displayed in gray. The node table records every assignment and the separate dictionary records the matching terms; the classification did not affect network inclusion, edge weights, or community detection.

The co-citation graph initially selected the 120 most frequently cited references (frequency ≥ 15), required co-citation support ≥ 5, and used cosine similarity. DOI parsing was corrected in Python 3.12.14 with NetworkX 3.6.1, NumPy 2.3.5 and pandas 3.0.1; absent or ambiguous DOI candidates used structured bibliographic identifiers. One flagged identifier was excluded without replacement (Supplementary Table S6c). The resulting graph contained 119 nodes and 6963 edges; Louvain detection used resolution 1.15 and primary seed 31. The 492-edge top-five-per-node plus maximum-spanning-tree backbone was display-only. Robustness used 90 runs per network. For author keywords, resolutions 0.8, 1.0, and 1.2 were crossed with association-strength thresholds 0.7, 0.8, and 0.9 and seeds 20,260,903 + 97*k* (*k* = 0,…,9). For co-citation, resolutions 1.00, 1.15, and 1.30 were crossed with supports 4, 5, and 6 and seeds 20 + 11*k* (*k* = 0,…,9). Complete graphs—not display backbones—determined modularity and partitions.

The collaboration graph used WoS author strings without claiming person-level identity resolution. The 120 most frequent strings occurring in at least five records were eligible, an edge required at least two joint records, and no record exceeded the prespecified 50-author exclusion. The complete analysis graph contained 365 edges; a top-four-per-node plus maximum-spanning-forest display backbone retained 281. For the collaboration visualization, the 70 highest-publication-eligible strings were used to form an induced subgraph, after which its largest connected component was retained; this deterministic rule yielded 69 displayed nodes. Annual team size and distinct author-string counts used all primary-corpus records. Because full names, affiliations, and ORCIDs were insufficient for reliable disambiguation, no bridge-author, productivity, quality, or influence ranking is reported.

#### 2.2.3. Lexicon-Based Title Concept Occurrence and Emerging Trends

A predefined, non-exclusive dictionary was used to record the occurrence of 34 concept groups in publication titles, spanning measurement, sources, particle transformation, transport, soil physicochemical mediation, plants, microorganisms, fauna, co-contamination, biogeochemical cycling, biodegradable materials, remediation, and risk. This was a deterministic lexical matching procedure rather than a machine-learning or semantic topic-classification model: a concept was recorded when a title contained a term included in the corresponding predefined lexicon, and records could match more than one concept. Annual rates were expressed as title matches per 1000 records within each corpus. These measures are therefore interpreted as exploratory title-level concept-occurrence indicators, not as semantic topic assignments or topic-prevalence estimates. The manual audit described above evaluated corpus relevance, which requires human semantic judgment, rather than re-labeling deterministic title matches. Complete-year trends were evaluated in SciPy 1.16.2 with two-sided Kendall’s tau-b (asymptotic *p* values when ties precluded exact calculation) and Theil–Sen slopes with 95% confidence intervals. Benjamini–Hochberg adjustment was applied separately to the family of 34 concepts within each corpus-by-start-year analysis at *α* = 0.05. Tests first covered 2016–2025 and were repeated for 2017 and 2018 because early annual denominators were small. A concept was designated rank-based start-year-stable only when its adjusted trend was positive and significant in every corpus under all three start-year definitions. As a denominator-aware sensitivity analysis, annual concept counts were also fitted with log-linked Poisson regression using log annual corpus size as an offset. Poisson point estimates were examined with inflation-only quasi-Poisson, HC3 sandwich, lag-1 Newey–West, and negative-binomial sensitivity specifications; two-sided Wald *p* values were adjusted over the same 34-concept family within each corpus-by-start-year analysis. Calendar year was centered within each 8–10-year series, and 2026 was excluded. Equations and complete output are supplied in the code and Supplementary Table S4b,c. These indicators quantify publication attention, not effect magnitude, causality, or evidence maturity; the short annual series limits variance and autocorrelation estimation.

Document-type dependence was assessed without changing the mixed-document primary corpus. Records indexed as Article or Article; Early Access formed an article-only subset (*n* = 2756). For the twelve start-year-stable title concepts, rates per 1000 article records were recomputed for complete years, and Kendall/Theil–Sen tests were repeated from 2016, 2017, and 2018 with Benjamini–Hochberg correction. The co-citation graph was also rebuilt under the same 120-reference, frequency ≥ 15, co-citation support ≥ 5, cosine-similarity, and Louvain settings used for the primary corpus; overlap in eligible nodes and in the twenty most frequently co-cited anchors was used as the principal structural sensitivity check. The article-only subset is interpreted as a robustness analysis, not as a methodologically superior replacement corpus (Supplementary Table S10).

Kleinberg burst detection was retained only as an exploratory auxiliary analysis for complete years 2016–2025. The primary parameters were *s* = 2.0 and *γ* = 0.5, with a nine-setting sensitivity grid crossing *s* = 1.5, 2.0, and 2.5 with *γ* = 0.4, 0.5, and 0.6. Because burst calls are parameter-dependent and the annual series is short, burst detection was not used to define stable temporal trends, rank research fronts, or support mechanistic conclusions. Detailed parameter results are reported in Supplementary Table S4d. Records from incomplete 2026 were excluded from formal trend and burst estimation and are shown descriptively only.

### 2.3. Critical Evidence Synthesis

Bibliometric maps were used to describe the development and organization of the literature; they were not treated as evidence of environmental effects. The critical synthesis was limited to terrestrial or soil-linked experimental, field, and review studies and followed the same causal sequence used throughout the manuscript: source and initial particle assemblage; retention, transport, aging, and fragmentation; changes in soil physical and chemical conditions; responses of plants, microorganisms, and fauna; and implications for risk and management. Marine and freshwater studies were used only when they explained the historical development of methods or transport concepts relevant to soil.

Studies entered the critical mechanistic synthesis purposively from the screened soil corpus, its cited-reference network, and reference chaining when they directly represented a link in the exposure-to-function sequence or a documented point of disagreement. This was not a second exhaustive screening census. Supplementary Table S9 covers all 75 unique sources cited in Section 4 and records study type, particle and exposure descriptors, endpoints, evidence-chain position, inclusion rationale, and verification source. Descriptors were checked against retained full text where available, matched local WoS title/abstract records, or the supplied bibliographic snapshot; source limitations are identified. Publication types are supported by the recorded sources, including six confirmations retained from the prior publication-type audit. NA denotes not applicable, NR requires absence after checking both full text and Supplementary Material, and NV denotes not verified from available evidence. Partial information is retained without inferring missing values. Model outputs and evidence from foliar, seed, or sediment systems are distinguished from direct soil-exposure measurements. Supplementary Table S8 states the qualitative claim boundaries; no dual-reviewer PRISMA flow, pooled effect size, screening-performance statistic, or reviewer-agreement statistic is claimed.

Evidence was interpreted by asking whether each study measured the successive links needed for its conclusion: the particles actually observed, their fate and contact, the altered soil or biological process, the functional consequence, its persistence or recovery, and the uncertainty surrounding extrapolation. Primary experimental or field evidence and secondary review evidence were recorded separately in Supplementary Table S9. Reviews are used as contextual or pathway-level synthesis, not as direct proof that an experimental link was measured. Recovery and quality control, causal attribution, environmental relevance, field persistence, and management trade-offs were evaluated separately. Figure 1 integrates these elements by showing corpus construction (Figure 1a), the distinct retrieval, inference, and sensitivity layers (Figure 1b), and the exposure-to-function sequence used to organize the review (Figure 1c); Table 1 states the corresponding analytical roles and limits. Section 4.2 then defines the claim-to-evidence boundary. The framework is qualitative and does not assign maturity scores, risk grades, or effect sizes.

The manual relevance judgments were made by the author.

## 3. Bibliometric Results

Publication and scope analyses establish the corpus trajectory; collaboration and co-citation describe the research context; and author-keyword and temporal analyses identify changes in the questions being asked. These bibliometric patterns are not treated as measures of environmental effect, causal importance, or evidence maturity.

### 3.1. Publication Growth and Corpus Selectivity

The query returned 5628 records within 2013–2026, increasing from 46 in 2018 to 228 in 2020, 692 in 2022, 1108 in 2024, and 1221 in 2025 (Figure 2a). The focus-anchored primary corpus followed the same trajectory, with 25, 120, 365, 659, and 729 records in those years. By 3 September 2026, 996 records had been retrieved, and 605 met the primary definition; both are incomplete-year counts and were neither annualized nor extrapolated.

Scope retention rose from 54.3% in 2018 to 59.7% in 2025 and 60.7% in partial 2026 for the primary corpus, while the broad and title-strict definitions bounded the 2013–2026 evidence base at 4787 and 2375 records (Figure 2b,c). This pattern means that an increasing share of retrieved records satisfied the prespecified focus anchors; it is not a human-validated estimate of semantic precision. The sensitivity range quantifies dependence on the screening definition. Dependence on the search term ground was not isolated because the frozen export does not identify which query term triggered each retrieval. The 2026 count is not interpreted as a decline because it represents an incomplete indexing interval under the same screening logic.

The manual relevance audit judged 115 of 120 sampled primary-corpus records to be in scope (95.8%; 95% Wilson interval 90.6–98.2%); five records that were flagged as ambiguous or partially off-scope were conservatively treated as not in scope. Because the audit sampled retained records only, this result is a relevance check on the included corpus rather than an estimate of recall, and the frozen 3200-record corpus was not redefined after the audit.

### 3.2. Collaboration Patterns

Figure 3 provides collaboration context rather than an explanation for divergent soil effects. In Figure 3a, node size encodes record frequency, color encodes mean publication year, and the adjacent list identifies the most frequent database strings; Figure 3b,c show annual team size and distinct author-string counts. The complete graph contained 120 recurrent WoS author strings and 365 eligible co-authorship links, of which 281 formed the display backbone; the 69-node connected display in Figure 3a follows the deterministic filter defined in Section 2.2.2. Distinct annual author strings increased from 519 in 2020 to 1731 in 2022 and 3584 in 2025, while median team size rose from five in 2020–2021 to seven in 2025 and partial 2026. These patterns indicate wider participation and larger teams, but the strings do not identify unique people and cannot support leadership, bridge, productivity, quality, or influence rankings.

### 3.3. Co-Citation Structure

Figure 4 locates the intellectual context of soil-microplastic research. Figure 4a displays the co-citation backbone and the most frequently co-cited references; Figure 4b identifies the journals contributing the most primary-corpus records, and Figure 4c reports community number and modularity; slight horizontal jitter in Figure 4c only separates coincident values. The complete 119-node co-citation graph contained 6963 edges. Across 90 complete-graph robustness runs, detected communities ranged from 2 to 17 and modularity *Q* from 0.069 to 0.109. Community number and modularity therefore varied substantially across the tested settings, and no single partition was privileged. The graph’s dense connectivity supports a shared co-citation core, but does not support the claim that the discipline objectively lacks subfields.

Frequently co-cited anchors connect terrestrial effects, analytical detection, soil occurrence, transport, and global plastic production. Their joint use is consistent with soil studies drawing on broader microplastic methods and exposure concepts for aggregates, pore networks, roots, rhizospheres, fauna, and agricultural management, while journal profiles place the primary corpus at the intersection of polymer pollution, soil processes, and biological effects. Co-citation records show shared use only; they do not indicate agreement, quality, effect direction, causal importance, or evidence maturity. Mechanistic conclusions were therefore based on particle characterization, soil conditions, exposure design, endpoints, and uncertainty rather than network position.

The article-only co-citation sensitivity analysis retained 112 of the 119 primary-corpus co-citation nodes (node-set Jaccard similarity = 0.889) and all 20 of the most frequently cited anchors. After the same citation-link exclusion, the article-only graph contained 119 nodes and 6473 edges compared with 6963 edges in the mixed-document primary graph. Reviews therefore affect edge density and some peripheral anchors, but their exclusion did not materially change the interpretation of a shared co-citation core (Supplementary Table S10).

### 3.4. Author-Keyword Networks

After removal of the search anchors, the author-keyword network contained 107 nodes and 216 eligible edges; 167 edges were retained for display (Figure 5a). In Figure 5a, node size represents keyword frequency, color identifies the prespecified mutually exclusive display domain, and the adjacent list reports frequent terms. Its frequent terms—microbial community, nanoplastics, biodegradable microplastics, heavy metals, polyethylene, soil properties, soil health, oxidative stress, biochar, and cadmium—connect material identity and transformation with fate, soil processes, biotic responses, co-contaminants, and management.

The primary Louvain solution contained 13 communities (*Q* = 0.697), but robustness yielded 9–14 communities (*Q* = 0.692–0.708). Figure 5c reports the 90 parameter–seed combinations; slight horizontal jitter only separates coincident points. These values show a modular co-occurrence graph under the tested definitions, whereas the changing community count cautions against treating one partition as unique; node-level partition stability was not quantified. Figure 5 therefore uses a deterministic display-domain assignment for color, with unmatched terms shown in gray and reports the algorithmic community range separately; these display domains are not inferred communities.

Under the declared display dictionary, the largest sums of author-keyword occurrences concerned plants, microorganisms, and fauna; particle identity and transformation; and risk or management (Figure 5b). Fate/exposure, soil properties/cycles, and co-contaminants remained connected rather than isolated. These totals cover the six named domains and exclude the gray Other linked terms category; they describe the figure’s organization rather than the prevalence or maturity of mechanistic evidence. The network is nevertheless compatible with the manuscript’s sequence: define the particle, establish its fate and contact, and only then interpret soil function or management.

Keyword frequency and mean year quantify attention, not causal importance. A recent term can represent an emerging question supported mainly by short experiments, whereas a long-standing analytical limitation can remain unresolved. For this reason, network topology is interpreted together with the direct evidence chain in Section 4.

### 3.5. Complete-Year Title-Concept Occurrence Trends

The rank-based analysis identified twelve title concepts with positive Benjamini–Hochberg-adjusted trends in all three corpora when the complete-year start was moved from 2016 to 2017 and 2018; no concept met an equivalent stable-decrease criterion (Figure 6). Median Theil–Sen slopes across the nine scope-by-start combinations were largest for plant/crop performance (+18.33 records per 1000 per year), biodegradable plastics (+11.58), and aging/weathering (+6.05). Biochar (+4.98), remediation/removal (+4.42), carbon cycling (+4.18), greenhouse-gas flux (+4.07), co-contamination (+3.34), nitrogen cycling (+3.09), risk assessment (+3.05), additives/leachates (+2.94), and biodegradation (+2.32) also met the rank-based criterion. All twelve had positive offset-Poisson slopes in every design, but statistical significance depended on the variance model: seven remained adjusted-significant in all nine designs under quasi-Poisson inference, and only co-contamination and nitrogen cycling passed every evaluated robust specification. The twelve are therefore treated as directionally stable signals rather than universally significant trends. Microbial community, heavy metals, rhizosphere, omics, and biofilm/plastisphere passed the original rank-based start but not every alternative start.

Together, these lexicon-matched increases are consistent with a broad shift from occurrence and analytical methods toward conditional questions about particle transformation, soil and biotic responses, biogeochemical cycling, and management. Greater title-concept visibility does not demonstrate that mechanisms are resolved or that effects persist under field conditions.

The article-only temporal sensitivity analysis showed the same directional result: all twelve start-year-stable concepts retained positive Theil–Sen slopes when testing began in 2016, 2017, or 2018. Ten of the twelve remained Benjamini–Hochberg-significant and positive for all three start-year tests; the two concepts additives/leachates and remediation/removal remained positive but did not meet the adjusted-significance criterion for every start year. The sensitivity analysis is therefore interpreted as support for the direction of the principal title-concept occurrence shifts, not as evidence that document types have identical growth rates (Supplementary Table S10).

The heat map standardizes each of the twelve start-year-stable concepts within its own annual history, so color identifies relative change rather than cross-concept abundance (Figure 6). The gray-outlined 2026 column contains the incomplete interval through 3 September and was excluded from formal trend testing. Because the concepts are non-exclusive lexical occurrence indicators, the colors do not encode effect size, effect direction, causality, or evidence maturity. Absolute rates, scope-specific tests, and start-year sensitivity results are provided in Supplementary Tables S4–S6 and the analytical audit workbook. This separation prevents a rapidly growing but infrequent title concept from being mistaken for the largest or most mature evidence domain.

### 3.6. Trend Robustness and Recent Title-Concept Profile

Complete-year trend testing and incomplete-year description were deliberately separated (Figure 7). Figure 7a compares median Theil–Sen slopes across the three corpus definitions and the 2016, 2017, and 2018 start-year choices; its symbols distinguish the three levels of offset-model robustness. Across all three scope definitions and all three start-year choices, the strongest directionally stable increases concerned plant/crop performance, biodegradable plastics, and aging/weathering; carbon and nitrogen cycling, co-contamination, biochar, remediation, and risk assessment formed a second set of increasing signals. Aging/weathering, biochar, biodegradable plastics, co-contamination, nitrogen cycling, remediation/removal, and risk assessment also remained adjusted-significant in all nine quasi-Poisson analyses; only co-contamination and nitrogen cycling were retained by every robust variance specification. Each title concept is interpreted only as a change in the lexical emphasis of publication titles, not as evidence that the corresponding environmental effect is stronger or more certain.

As an explicitly exploratory auxiliary check, Figure 7c shows that co-contamination produced the most reproducible 2025 burst: it was detected in six of nine settings in the primary corpus and all nine settings in the title-strict corpus under each tested year basis. This result is not used to define a research frontier or to support a mechanistic conclusion. Biochar generated a 2025 burst in only three of nine settings despite a directionally stable complete-year trend. Full burst parameter results are provided in Supplementary Table S4d.

Figure 7b shows that, within the 605 primary-corpus records indexed by 3 September 2026, the highest title-match rates per 1000 records concerned plant/crop performance (191.74), sources/inputs (107.44), biodegradable plastics (100.83), microbial community (100.83), heavy metals (95.87), toxicity/oxidative stress (85.95), transport/migration (82.64), and soil fauna (71.07). These values describe the composition of an incomplete indexing interval; they are not calendar-matched changes and were not used to assert annual growth, burst onset, effect magnitude, or evidence maturity.

Biodegradability, biochar, heavy metals, greenhouse gases, plants, and microorganisms are considered only where they modify or reveal links in the exposure, fate, or functional effects of soil microplastics.

Together, Figure 5, Figure 6 and Figure 7 identify where research attention moved—from occurrence and detection toward particle transformation, effective contact, soil and biological processes, and conditional management questions. They do not establish that the resulting evidence is comparable, causal, persistent, or decision-ready. Section 4 therefore evaluates these same signals mechanistically by asking which exposure-state and exposure-to-function links were actually measured.

## 4. Mechanistic Synthesis

### 4.1. Exposure State as the Comparison Unit

Studies of soil microplastics often examine non-equivalent exposures. Cross-study comparison is more defensible when five descriptor groups are reported together: material identity (polymer and additives), particle attributes (size, shape, and surface state), exposure history (source, applied amount, aging, and prior transport), soil context (texture, structure, chemistry, water regime, organic carbon, and relevant biota), and spatiotemporal observation context (sampling depth, exposure duration, and timing of measurement). Concentration remains essential, but it cannot substitute for the particle, soil, and observation information needed to compare studies. Exposure non-equivalence is evaluated as one possible source of heterogeneity rather than a complete explanation for every disagreement.

Sources first define an initial particle assemblage. Weathering, fragmentation, mineral coatings, biofilms, retention, preferential flow, and management then alter particle distribution and the surfaces or additives available for interaction [12,13,14,15]. Two studies using the same mass concentration may therefore expose roots or microorganisms to different particle numbers, surface areas, chemical burdens, or contact durations.

Causal attribution is strengthened when changes in particle state are linked to altered soil conditions or receptor contact and then to prespecified functional endpoints. Changes in microbial composition, plant growth, nutrient turnover, or greenhouse-gas flux are responses under specific exposure conditions, not inherent properties of microplastics as a class. The relevant task is to determine which link changed and under which conditions the response remained consequential.

The complete-year shift toward polymer transformation, crop performance, co-contamination, biogeochemical processes, and management shows where publication attention moved along the exposure-to-function chain; it does not show that downstream evidence matured at the same rate. Particle recovery defines the observed fraction, fate and contact measurements connect that fraction to a receptor, and process measurements strengthen causal interpretation. Evidence for persistence requires longitudinal observations or repeated sampling with appropriate controls, whereas management claims additionally require comparisons of efficacy, feasibility, trade-offs, and unintended effects. None of these links can be inferred from title-concept occurrence frequency alone.

### 4.2. Cross-Study Comparability

Five descriptor groups shape cross-study comparability (Figure 8): material identity, particle attributes, exposure history, soil context, and spatiotemporal observation context. Figure 8a defines these groups, Figure 8b lays out the successive inferential links, and Figure 8c groups the minimum design requirements for measurement comparability, causal attribution, field transfer, and decision evaluation. Individual reporting descriptors such as polymer, size, shape, recovery, soil type, and duration have appeared in previous methodological reviews [33,34,35]; they are not presented here as individually novel. The added step is to integrate them into an exposure-state representation and then apply an explicit claim-to-evidence boundary. The observation gate asks whether recovery, size cutoffs, units, and polymer-confirmation methods measured the same particle fraction; the inference gate asks whether endpoints and claim scope stop at the same link, from fate and contact to process, function, persistence, or management. Interpretation should account for differences in these descriptor groups, analytical recovery, endpoints, and timescale before results are treated as directly comparable. The framework is qualitative rather than a maturity score, risk grade, threshold, or effect-size model. Table 2 specifies the evidence required at each claim level.

A study supports only the conclusion corresponding to the link it measured. Particle characterization identifies the observed material; retention and transport measurements establish fate; localization at receptor interfaces documents contact, whereas root, microbial, or faunal endpoints quantify responses; soil-process measurements support functional interpretation; and longitudinal observations with appropriate controls evaluate persistence or recovery. Moving from one link to the next requires additional evidence rather than stronger wording.

This diagnostic makes many apparent contradictions scientifically interpretable. Results obtained with different analytical recovery, particle states, soils, endpoints, or durations should first be treated as conditional observations rather than direct replications that disagree. Likewise, an association between particles and a biological response does not identify whether the operative pathway was the particle surface, an additive, a degradation product, a co-contaminant, or a particle-induced change in soil structure.

The framework does not imply that every disagreement can be reconciled, and this review does not quantify the proportion attributable to each source of non-equivalence. Experimental error, selective reporting, and endpoint choice may also contribute. It provides a practical test: account for analytical recovery, the five descriptor groups, endpoints, and timescale, then restrict interpretation to the last observed link. Its contribution is a bounded procedure for evaluating whether exposure non-equivalence can explain divergent findings and for matching mechanism, persistence, risk, and management conclusions to measured evidence.

### 4.3. Exposure-to-Function Mechanisms

Section 4.3 interprets the bibliometric signals in Figure 5, Figure 6 and Figure 7 through the exposure-to-function sequence. Each subsection separates observation from inference, explains how exposure conditions may alter outcomes, and identifies the evidence required for the next level of conclusion. Throughout Section 4, primary experimental or field studies are used to support only the links and endpoints they directly measured, whereas review and meta-analytic sources provide contextual or pathway-level synthesis; Supplementary Table S9 records each source’s evidence type and supported link, distinguishing measured endpoints from conditional model outputs.

#### 4.3.1. Measurement and Recovery

Analytical recovery determines which fraction of the soil microplastic assemblage is observed. Soil minerals and organic matter complicate extraction and visual sorting [38,39]. Recovery can be tested with defined flotation protocols, but performance depends on polymer density and particle size [40,41]. Spectroscopic and hyperspectral approaches can support calibrated concentration estimation or polymer classification when models and libraries are independently validated [42,43], whereas total organic carbon–solid sample combustion provides a mass-oriented estimate after matrix removal [44]. Counts, mass, size distribution, and polymer composition are therefore method-conditioned observations rather than interchangeable exposure measures.

Comparable reporting is as important as analytical throughput. Studies should state sampled soil mass and depth, field replication, contamination controls, recovery tests using multiple polymers and sizes, the density-separation solution, digestion conditions, the lower detection limit, polymer-confirmation criteria, units, and treatment of fibers. Counts, mass, and size distributions answer different questions and should not be converted without explicit assumptions. Hyperspectral and spectroscopic screening can improve throughput, but automated classification requires validated libraries and independent error assessment [42,43].

An additional challenge is that analytical objectives differ. Spectroscopic imaging prioritizes polymer-resolved counts and morphology, thermal methods prioritize polymer mass, and emerging combustion or bulk-carbon approaches prioritize throughput and mass estimation. These outputs are complementary rather than interchangeable. Method selection should therefore follow the question: source apportionment may require polymer identity and particle morphology, transport studies require size distributions and recovery, and risk studies require exposure-relevant number, mass, surface area, and chemical information. A minimum reporting framework should make these differences explicit so that future meta-analyses do not combine incompatible metrics as though they represented the same exposure.

Nanoplastics create a sharper version of the same measurement problem. Agricultural-soil reviews emphasize that conventional density separation, microscopy, and routine spectroscopic workflows lose sensitivity as particle size approaches the nanoscale, while isolation from mineral and organic colloids, size-resolved recovery, polymer confirmation, and discrimination from natural nanoparticles remain difficult [45,46]. Consequently, a lower measured microplastic mass after aging or degradation cannot be interpreted as disappearance if smaller fragments may have crossed the analytical size cutoff. In this review, nanoplastic studies are used only where they clarify transformation, contact, or analytical limitations relevant to the exposure-to-function chain, and are not treated as direct evidence for all microplastic size classes.

#### 4.3.2. Source-Defined Particle States

Agricultural sources introduce microplastic assemblages that differ in polymer composition, geometry, weathering, additives, and input history. Incomplete recovery of plastic mulch leaves fragments that weather during cultivation [47], while sewage sludge introduces a distinct waste-derived particle mixture [5]. An agricultural review identifies additional source, fate, and effect pathways [48]; field evidence shows land-use contrasts [49]; farmland surveys identify source and pathway patterns [50,51]; and a duration-specific study reports accumulation under plastic mulching [52]. A broader freshwater–terrestrial review situates these sources within the development of microplastic research [53]. These inputs are not interchangeable before soil mediation begins.

The main limitation is attribution. Fields with longer mulching histories may also differ in irrigation, tillage, fertilizer use, crop rotation, and soil organic matter. Cross-sectional comparisons therefore cannot by themselves prove that microplastics caused every observed difference. Stronger evidence requires chronosequences with management records, paired fields, before–after designs, or repeated measurements that separate plastic exposure from broader intensification. Measurements should include particle abundance and mass, polymer composition, size and shape, aging state, vertical distribution, and relevant soil physical, chemical, and biological indicators. Such designs would strengthen attribution of soil-function changes to microplastic exposure; estimating risk would still require exposure–response and receptor-sensitivity evidence.

Long-term evidence is particularly valuable because repeated agricultural inputs can produce legacy effects that short incubations cannot represent. However, duration alone is insufficient: a field history must be connected to input records, recovery efficiency, polymer composition, and soil-profile sampling. Where these data are available, changes in aggregation, microbial biomass, nutrient supply, or crop performance can be evaluated as plausible functional consequences rather than assumed from surface abundance. The strongest future field studies will therefore integrate inventories with management chronology and process measurements, allowing source attribution and soil-quality interpretation to be tested within the same design.

Biodegradation or fragmentation does not automatically imply environmental safety. In the soil-microplastic literature considered here, the dominant biodegradable-plastic evidence concerns manufactured polymers and agricultural materials such as PLA, PBAT, PBS/PBSA, PHA, and blends rather than naturally occurring polymer debris [54,55,56,57,58,59,60,61]. “Biodegradable” is also not synonymous with “natural” or “bio-based”: some biodegradable polymers are bio-based, others are fossil-derived, and their degradation pathways and products differ. A lower recovered parent mass may coexist with smaller micro- or nanoplastic fragments, soluble oligomers, additives, or transient carbon inputs; the next level of claim therefore requires mass balance, product identification, and functional measurements rather than disappearance of the parent material alone [60].

#### 4.3.3. Particle Transformation and Soil Transport

Transport and aging alter both the distribution and surface properties of microplastics in soil. Transport depends on soil texture, aggregation, preferential flow, ionic strength, dissolved organic matter, and particle size, density, shape, and surface charge [11,13,15]. Runoff, flooding, and water erosion can redistribute particles beyond an application area [12,13,62]. Wind erosion is a documented terrestrial pathway [8], while tillage, irrigation, and bioturbation can provide other lateral or vertical pathways [14,15]. Consequently, detection at depth reflects a coupled particle–soil history rather than mobility as an intrinsic polymer property.

Aging adds a second dimension. Oxidation, cracking, biofilms, mineral coatings, and natural organic matter change density, surface charge, hydrophobicity, and contaminant interactions. Experiments with pristine commercial particles therefore provide only a starting point. Future work should compare pristine and field-aged particles, track mass recovery, and distinguish irreversible retention from temporary attachment or fragmentation below detection. Movement to surface water is only one pathway [8,12]. Wind-driven transport is documented in terrestrial settings [8], and trophic transfer has been demonstrated or reviewed for terrestrial food chains [63,64]. Deep-soil storage and plant-surface retention remain plausible, but require pathway-specific field evidence before their relative importance can be quantified.

Transport should also be framed as a mass-balance problem. Detection at depth does not alone identify the pathway, and non-detection may reflect retention, degradation, fragmentation below the analytical cutoff, or incomplete recovery. Studies that report only the fraction leaving a laboratory column cannot establish landscape-scale export. More defensible designs will quantify input, retained mass, effluent, particle-size change, and uncertainty; combine intact soil cores with field monitoring; and distinguish advective transport from bioturbation, tillage redistribution, and atmospheric re-emission. This approach would connect migration studies to exposure assessment rather than treating movement as an isolated material property.

#### 4.3.4. Contact Pathways and Soil Functions

Microplastics can change aggregation, pore structure, bulk density, water availability, nutrient distribution, and microbial habitat, thereby altering conditions experienced by roots and soil organisms [16,20,65]. Plant and root responses have been reported under specific soil treatments [25,26,27]. Evidence from foliar exposure [66] is not used to support soil-mediated effects and is retained only to show why the exposure route must be separated. A seed-capsule study identified delayed germination and root growth [67]. Microbial and faunal responses depend on treatment design [29,54,68], and polymer-specific microbial responses have also been reported [69]. Polymer, size, surface state, dose, soil, exposure route, and duration must therefore remain explicit.

The rhizosphere can mediate effects and, for some materials, accelerate transformation. Microbial colonization and soil incubation can transform biodegradable polymers [55,56,57]. One rhizosphere study reported accelerated breakdown [58]. Faster breakdown is not equivalent to complete mineralization or safety because it can generate smaller fragments, dissolved products, or transient microbial stimulation. Studies should therefore quantify mass loss, molecular-weight change, mineralization, fragment generation, degradation products, and biological response within the soil–root pathway.

A central unresolved issue is whether changes in community composition correspond to persistent changes in function. Amplicon sequencing can reveal taxonomic reorganization, and metagenomics or metabolomics can identify candidate pathways, but functional inference requires direct measurements of nutrient flux, enzyme activity, root nutrient acquisition, biomass allocation, and recovery after exposure. The increasing use of multi-omics is therefore most informative when embedded in a causal chain linking particle properties to soil conditions, microbial processes, and plant outcomes. Without this linkage, highly resolved molecular data may describe response without explaining ecological consequence.

#### 4.3.5. Carbon and Nitrogen Responses

Carbon and nitrogen responses occur downstream of exposure characterization and require source attribution and process measurements. Microplastics can alter decomposition-relevant microsites [70]. Reviews synthesize plausible carbon- and nitrogen-cycle pathways [22,71] and climate-relevant pathways [72], but these review sources are used here only as contextual synthesis. Direct mechanistic support is assigned to primary studies only for the endpoints they actually measured: a defined experiment reported altered nitrification and nitrogen-functional bacterial groups [73], and experiments report material- and soil-specific changes in carbon dioxide, nitrous oxide, or methane fluxes [23,24,74]. Fiber-specific responses [74] and divergent methane responses among paddy soils [75,76] demonstrate why particle and soil context must remain explicit. Recent primary studies connect rhizosphere conditions and microbial physiology to carbon sequestration or dissolved-organic-matter transformation [77,78], while direct experiments link microbial restructuring and organic-matter decomposition to carbon release under particular exposures [79,80]. A flux response alone does not identify the operative mechanism or carbon and nitrogen source (Supplementary Table S9).

Recent evidence reinforces this conditional interpretation. Aged polylactic-acid microplastics were associated with lower methane emissions in one paddy-soil study through identifiable process changes [76]. Polyethylene microplastics reduced methane emissions in acidic and alkaline paddy soils but produced no significant change in neutral soils [75], while a separate microplastic–biochar–earthworm experiment reported context-dependent CO_2_ and N_2_O responses [81]. A hydrochar co-treatment also produced a context-specific greenhouse-gas response [82]. In a separate study, PLA/PBS fabric, PBAT/pulp laminate, and PLA pieces produced material- and time-specific CO_2_ and N_2_O responses [59]. Effects on carbon sequestration and dissolved-organic-matter transformation have also been linked to rhizosphere silicon and microbial physiological adaptation [77,78]. These results cannot be combined into one global emission factor.

The same caution applies to carbon accounting. Biodegradable polymers can introduce an external carbon substrate, whereas conventional polymers may primarily modify physical habitat or microbial access to native organic matter. A change in CO_2_ flux can therefore reflect mineralization of polymer-derived carbon, priming or suppression of native soil organic matter, altered plant inputs, or a combination of these processes. Isotopic labeling, source-partitioning models, microbial carbon-use efficiency, and measurements of particulate and mineral-associated organic carbon are needed to separate these pathways. Without source attribution, a flux response cannot be interpreted reliably as soil-carbon loss or gain.

#### 4.3.6. Co-Contaminants and Carbonaceous Amendments

Co-contamination studies must separate particle, chemical, and amendment pathways. The co-treatment studies reviewed here used biochar, a carbon-rich product of biomass conversion under limited oxygen, and hydrochar, which is produced by hydrothermal carbonization, respectively [81,82]. Because the materials and production routes differ, their results should not be treated as interchangeable. They enter this review only as factors tested together with microplastics, not as substitute subjects or inherently beneficial interventions. Review evidence indicates that microplastic surfaces and aging can alter contaminant sorption, co-transport, and release [83,84], while a riparian-sediment experiment compared pristine and aged particles and reported changes in dissolved-organic-matter bioavailability and carbon emissions [85]; transfer of that specific mechanism to soil remains a hypothesis. Primary soil studies also show that vector effects are conditional rather than uniformly toxicity-enhancing: microplastic-mediated hydrophobic organic contaminant (HOC) bioaccumulation in earthworms depended on particle size and chemical diffusion [86], whereas another soil study found that microplastics delayed phenanthrene leaching and reduced its bioavailability to wheat [87]. Evidence at the nanoscale should be bounded even more carefully. An open-access, model-based study spanning 100 nm to 1 mm particles in agricultural-soil transport scenarios found that most organic contaminants desorb too rapidly for micro- or nanoplastics to substantially increase mobility; appreciable particle-facilitated transport was restricted to specific combinations of rapid flow and strongly hydrophobic contaminants [88]. Thus, neither smaller particle size nor sorption to a particle is by itself evidence of increased biological exposure. Factorial designs should compare contaminant-only, microplastic-only, amendment-only, and combined treatments and should measure dissolved or otherwise bioavailable contaminant fractions together with transport, receptor exposure, and soil-process endpoints.

#### 4.3.7. Exposure–Hazard Separation and Evidence Tiers

Risk assessment requires a broader evidence chain than hazard identification. Spatial risk mapping can organize information on sources, exposure, and sensitive receptors, but its reliability depends on comparable concentration data, transparent thresholds, and uncertainty treatment [89]. Current effect thresholds are often derived from short laboratory studies and may not match field particle mixtures or chronic exposure. A defensible soil risk framework should separate exposure magnitude and probability, particle and chemical hazard, ecological receptor sensitivity, and functional consequence. It should also report uncertainty arising from analytical recovery, size detection limits, spatial heterogeneity, and extrapolation among polymers and species. Without these steps, a risk index may appear quantitative while remaining strongly dependent on arbitrary scoring choices.

The distinction between hazard and risk is especially important for co-contamination. A laboratory interaction at a selected particle-to-contaminant ratio demonstrates a possible mechanism, but field relevance depends on co-occurrence, aging, soil chemistry, receptor contact, and persistence of the altered bioavailable fraction. Risk assessment should therefore use tiered evidence: analytical confirmation of exposure, mechanistic evidence for interaction, organismal or functional response at realistic conditions, and uncertainty-bounded extrapolation. This structure prevents a single sorption coefficient or high-dose toxicity test from being treated as a complete environmental-risk estimate.

### 4.4. Research Priorities

#### 4.4.1. Minimum Exposure-State Reporting

Analytical incompatibility is an immediate barrier to cumulative evidence. In addition to procedural blanks, matrix spikes, recovery, detection limits, and polymer-confirmation rules [33,34], every study should report a minimum exposure-state descriptor: polymer identity; particle count or mass; size and shape distribution; pristine, aged, or field-recovered status; known additives or co-contaminants; dose and input history; soil texture, organic carbon, pH, water regime, and sampling depth; exposure duration; and recovery uncertainty. The descriptor is not a score. Its purpose is to show whether two studies measured sufficiently similar states to justify comparison.

Methods should then be selected for the inference required. Total organic carbon–solid sample combustion can support mass-oriented quantification after matrix removal [44], whereas validated hyperspectral imaging can support polymer classification [43]. Neither output alone answers every question. Counts, mass, size distribution, polymer identity, morphology, surface condition, and chemical burden should be reported as distinct but linked descriptors and should not be converted without empirical calibration. Open spectral libraries, validated image classification, and machine-readable metadata would make those descriptors more reproducible.

#### 4.4.2. Persistence Across Time, Depth, and Sites

The second priority is to align exposure design with environmental history. A 25-year sewage-sludge study and a two-season European pedoclimatic experiment illustrate the value of long-duration and geographically distributed evidence [90,91]. Their contrasting designs underscore the need to distinguish application history from experimentally imposed exposure and to account for site context. Future field designs should combine baseline measurements, repeated sampling, known application records, depth-resolved inventories, and paired controls. Reporting uncertainty around mass recovery and spatial variance is as important as reporting the mean.

Crop studies are beginning to bridge laboratory and field conditions. A soil–crop study considered microplastic uptake and crop impacts under exposures described as realistic [92]. It is used here as qualitative uptake context; specific particle-size or dose effects are not assigned without verified exposure descriptors (Supplementary Table S9). Multi-site experiments across soil textures, crops, climates, and management systems, using harmonized materials and endpoints, would provide a stronger basis for identifying when effects are general and when they are site-specific.

#### 4.4.3. Particle-State Links to Soil Processes

Mechanistic studies now generate increasingly detailed microbial, molecular, and physiological data, but the field must avoid equating resolution with causality. A recent meta-analysis found that responses of soil microbial diversity and community structure vary with experimental conditions and are not uniformly large [93]. This reinforces the need to specify the causal chain being tested. Factorial designs should separate particle structure, polymer chemistry, additives, aging products, and co-contaminants; mediation analyses should be supported by temporal or manipulative evidence; and molecular observations should be linked to measured process rates.

Within the Figure 8 inferential chain, observation of a changed particle state or soil exposure does not, by itself, establish fate/contact, an altered process, functional consequence, persistence/recovery, or management benefit. Each transition requires its own measurement, control, and timescale. The chain is therefore used to calibrate claim scope rather than to create a second classification or a numerical maturity score.

#### 4.4.4. Net Risk and Management Outcomes

Translation requires indicators that are both scientifically defensible and operationally measurable. Particle abundance alone is inadequate because identical counts can represent different sizes, polymers, surface areas, chemical burdens, and biological access. A tiered risk framework should begin with exposure characterization, then evaluate particle and chemical hazard, receptor sensitivity, soil-function consequence, and uncertainty. Thresholds should not be transferred among polymers or soils without testing the assumptions that make them comparable. Spatial models are useful for prioritization, but their outputs should be presented as uncertainty-bounded screening results rather than precise estimates of ecological damage.

Management evaluation should compare prevention, collection, substitution, and remediation across the full material lifecycle. Biodegradable mulch may reduce persistent-film residues under some conditions, yet biodegradability does not by itself establish environmental benignity. The studies synthesized here predominantly concern manufactured biodegradable polymers and agricultural materials rather than naturally occurring polymer debris, and bio-based origin must be distinguished from biodegradability [60,61]. Degradation rates and products depend on soil, climate, thickness, polymer formulation, and management, and can include smaller fragments, oligomers, additives, or altered carbon inputs [60]. Likewise, remediation that immobilizes particles may reduce immediate mobility without removing the source. Future assessments should therefore combine agronomic performance, plastic recovery, fragment generation, soil-function endpoints, greenhouse-gas consequences, economic feasibility, and unintended effects. This broader evidence base is necessary for recommendations that remain valid outside a single laboratory system.

### 4.5. Limitations

WoSCC was used as the sole bibliographic source to maintain one indexing framework and may underrepresent regional journals, books, reports, or records indexed elsewhere. The exported batch files identify the Web of Science Core Collection but do not preserve the selected constituent-index combination as a record-level field; this limits exact reconstruction of interface settings. The term ground increases recall but retrieves peripheral cross-media literature. Deterministic focus anchoring and two alternative sensitivity corpora reduce scope dependence. A year-stratified manual relevance audit of 120 retained primary-corpus records was added in revision and found 115 in scope under the prespecified relevance question; however, it used one evaluator, sampled retained records only, and therefore does not estimate recall or inter-reviewer agreement. Precision, recall, F1, and kappa are not claimed.

The integrated export provides titles, abstracts, author keywords, cited references, languages, and document types across the full declared period. Remaining missingness is explicit: 3197/3200 records contain abstracts, 3046/3200 contain author keywords, and 3199/3200 contain cited references. Early annual denominators were small, so formal trend claims were restricted to concepts meeting the rank-based criterion across three corpora and three start-year definitions and were stress-tested with offset count models. All twelve slopes remained positive, but robust significance varied because each fit contained only 8–10 annual observations; these results support directional signals, not a claim of universal statistical significance. The article-only sensitivity analysis retained positive directions for all twelve principal title-concept occurrence trends and most primary co-citation anchors, but it is a robustness check rather than an alternative definition of the field. Author strings were not identity-disambiguated, node-level partition stability was not quantified, and the title-concept analysis is a deterministic lexicon-based occurrence analysis rather than semantic topic classification; it can miss conceptually relevant papers that do not use the predefined title terminology. The human audit therefore evaluates corpus relevance, not semantic topic-label accuracy. The 2026 interval is incomplete through 3 September. The review therefore supports conditional mechanisms and testable design priorities, not universal toxicity values, complete field representativeness, or decision-ready thresholds. For S9, full text was retained for only a subset of sources; unresolved conditions are explicitly identified. Publication-type confirmation is recorded separately from verification of experimental descriptors. The matrix is not a complete full-text systematic review. Targeted checks identified one flagged citation identifier; its inclusion–exclusion sensitivity is reported in Supplementary Table S6c. These checks do not validate every remaining citation linkage.

## 5. Conclusions

Reported effects of soil microplastics vary across studies that differ in polymer composition, particle size and shape, aging and additive profiles, soil properties, analytical recovery, exposure duration, and endpoint selection. These differences can alter the particle fraction detected, its transport and biological contact, and the soil process ultimately measured. Exposure non-equivalence is therefore a plausible contributor to inter-study heterogeneity, although it does not exclude experimental error, selective reporting, or genuine context-specific responses.

Bibliometric analysis of the 3200-record primary corpus showed a shift in publication emphasis from occurrence and detection toward plant and crop responses, biodegradable plastics, aging, biogeochemical cycling, co-contamination, and management. Twelve title concepts increased consistently across corpus definitions and start years, but only co-contamination and nitrogen cycling were significant under every robust model specification. The revision-stage relevance audit judged 115 of 120 sampled primary-corpus records in scope, and the article-only sensitivity analysis retained positive directions for all twelve principal title-concept occurrence trends together with 112 of 119 primary co-citation nodes. These checks reduce dependence on a single corpus boundary or document-type composition but do not convert deterministic lexical occurrence indicators into semantic topic-prevalence estimates. The findings therefore indicate changes in research attention rather than the magnitude or certainty of environmental effects.

To improve comparability, studies should report polymer and particle characteristics, analytical recovery, exposure history, soil context, sampling depth, and duration; directly measure transport or contact and the relevant soil process; and test persistence across time and sites. Risk and management recommendations should be limited to the links directly supported by evidence and should include treatment efficacy, soil-function outcomes, feasibility, and unintended effects. This approach would strengthen causal interpretation and the transfer of laboratory results to field conditions.

## Figures and Tables

**Figure 1:**
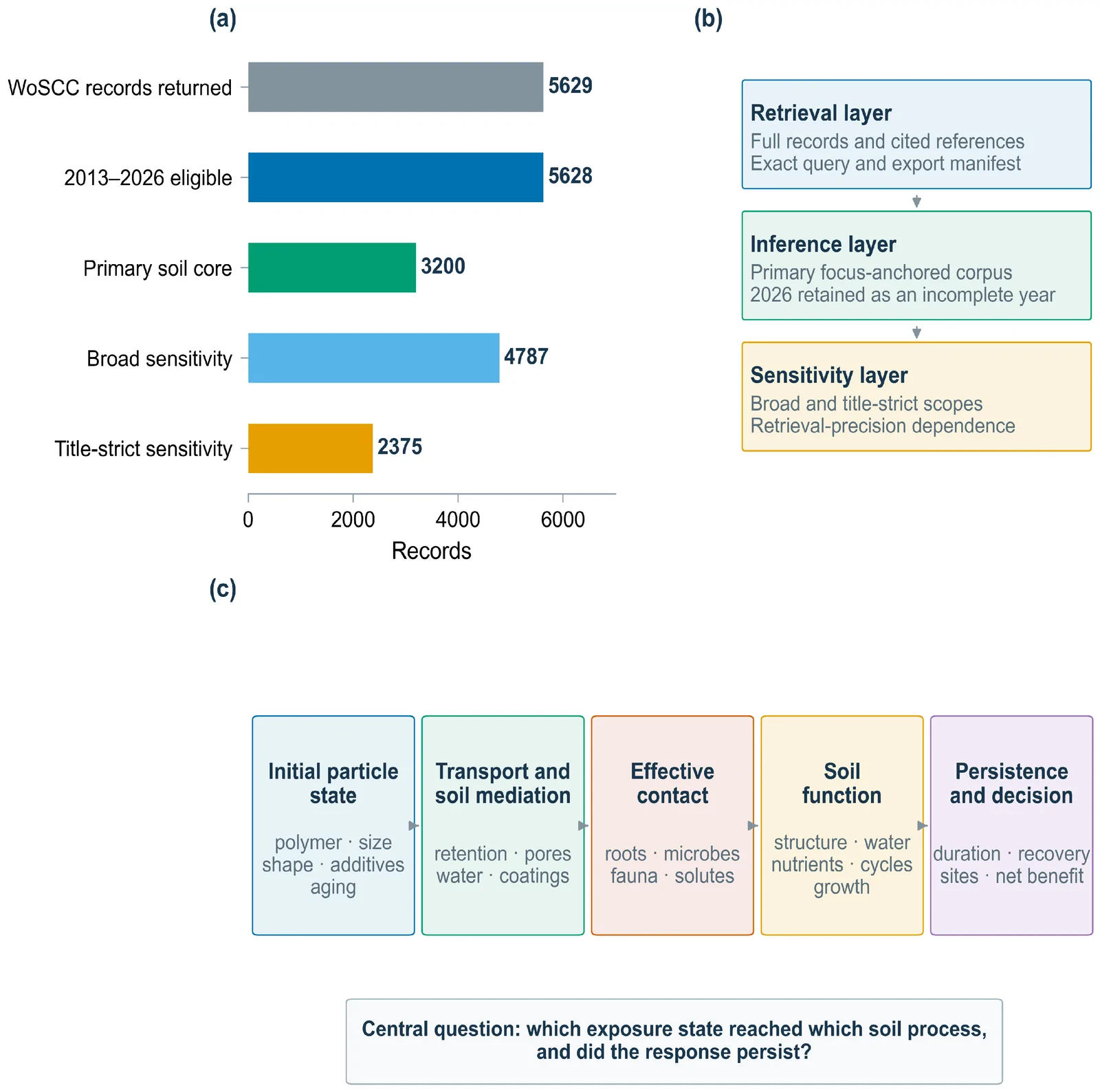
Corpus construction (**a**), analytical layers (**b**), and the exposure-to-function framework (**c**). WoSCC, Web of Science Core Collection.

**Figure 2:**
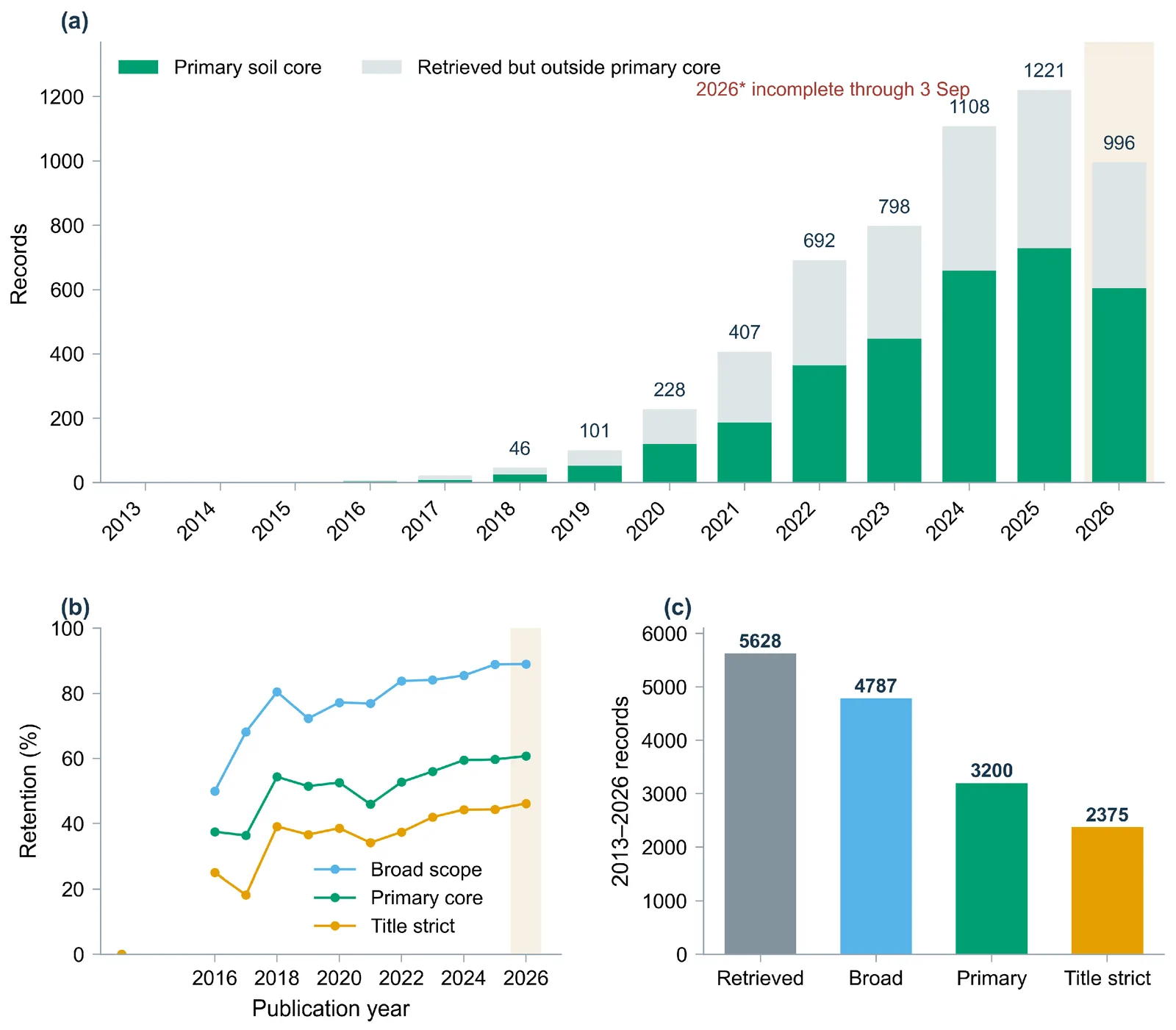
Annual publication trajectory (**a**), annual scope retention (**b**), and total corpus sensitivity (**c**), from 2013–2026. The asterisk and beige shading mark incomplete 2026 data through 3 September. Retention is undefined when no records were retrieved.

**Figure 3:**
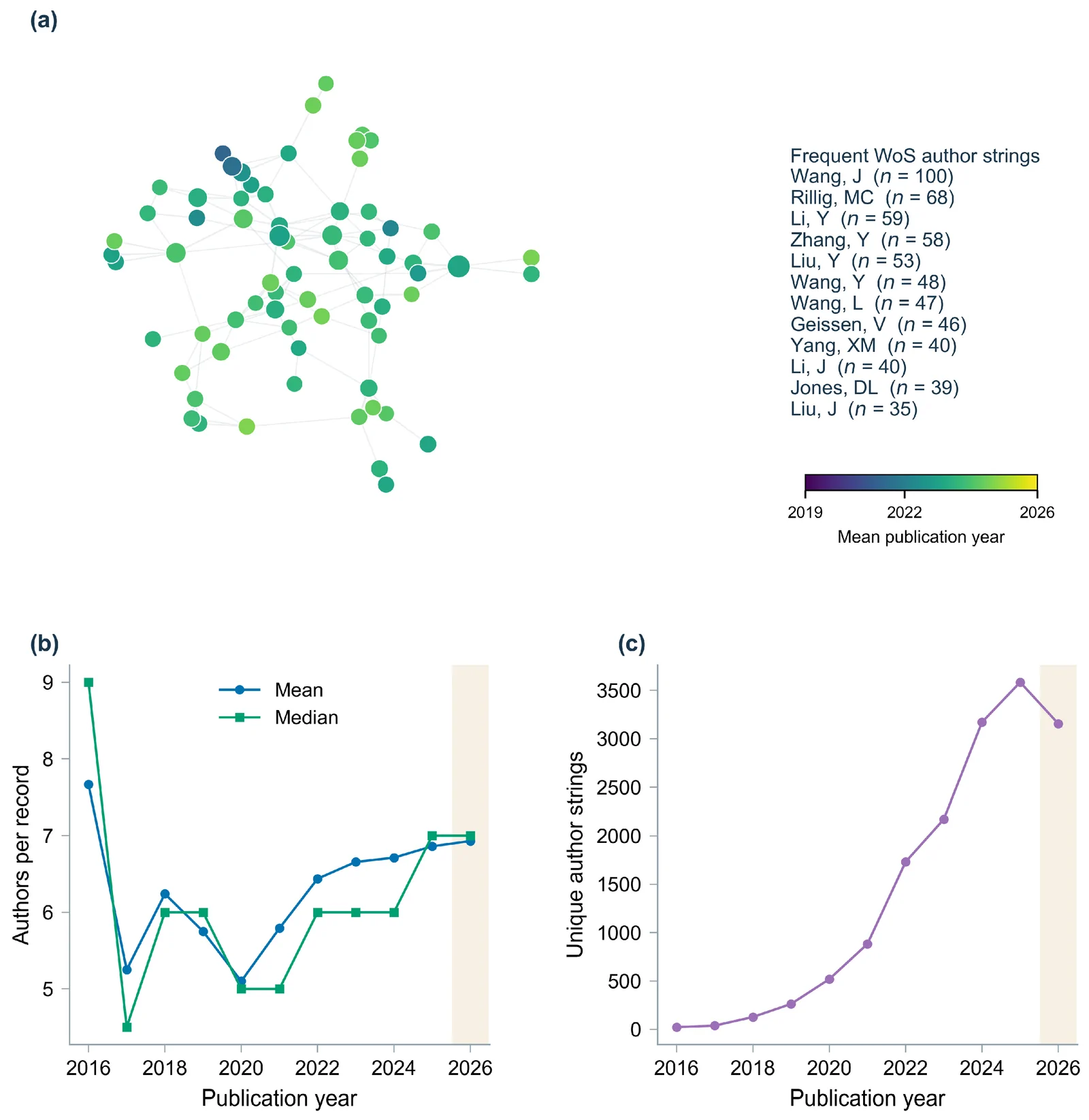
Co-authorship structure (**a**), annual team size (**b**), and annual author-string counts (**c**) in the primary corpus. In (**a**), node size and *n* indicate record frequency; color indicates mean publication year. Labels are database author strings, not citations. Beige shading marks incomplete 2026.

**Figure 4:**
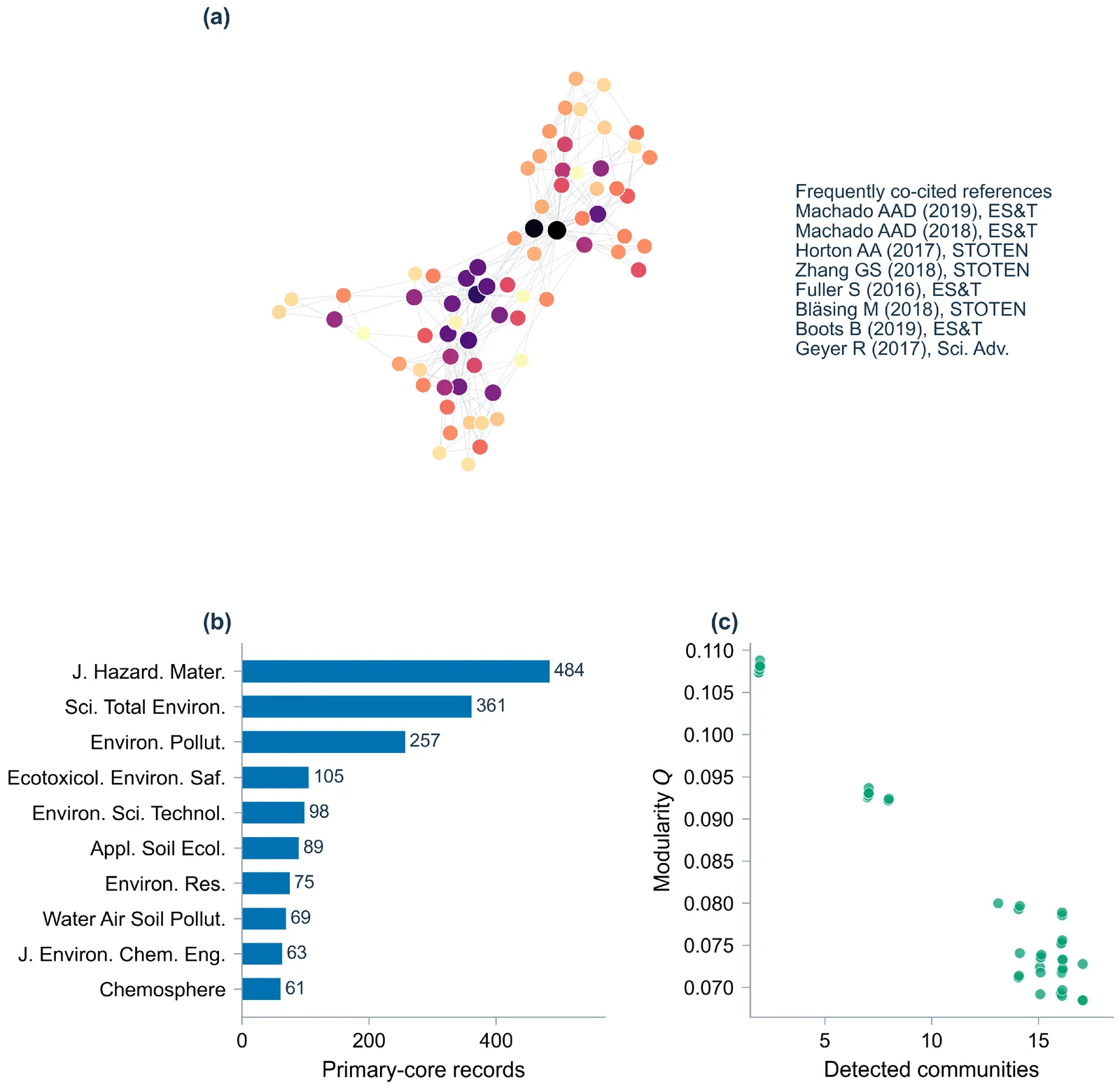
Co-citation network (**a**), leading source journals (**b**), and co-citation partition robustness (**c**). In (**a**), 70 nodes and 253 edges are displayed from the 119-node analysis graph. Node size and log-scaled color indicate citation frequency. Author–year labels are data identifiers, not manuscript citations (Figure4_label_key.csv). In (**c**), green points represent 90 parameter–seed combinations; *Q* denotes modularity. ES&T, Environmental Science & Technology; STOTEN, Science of the Total Environment; Sci. Adv., Science Advances.

**Figure 5:**
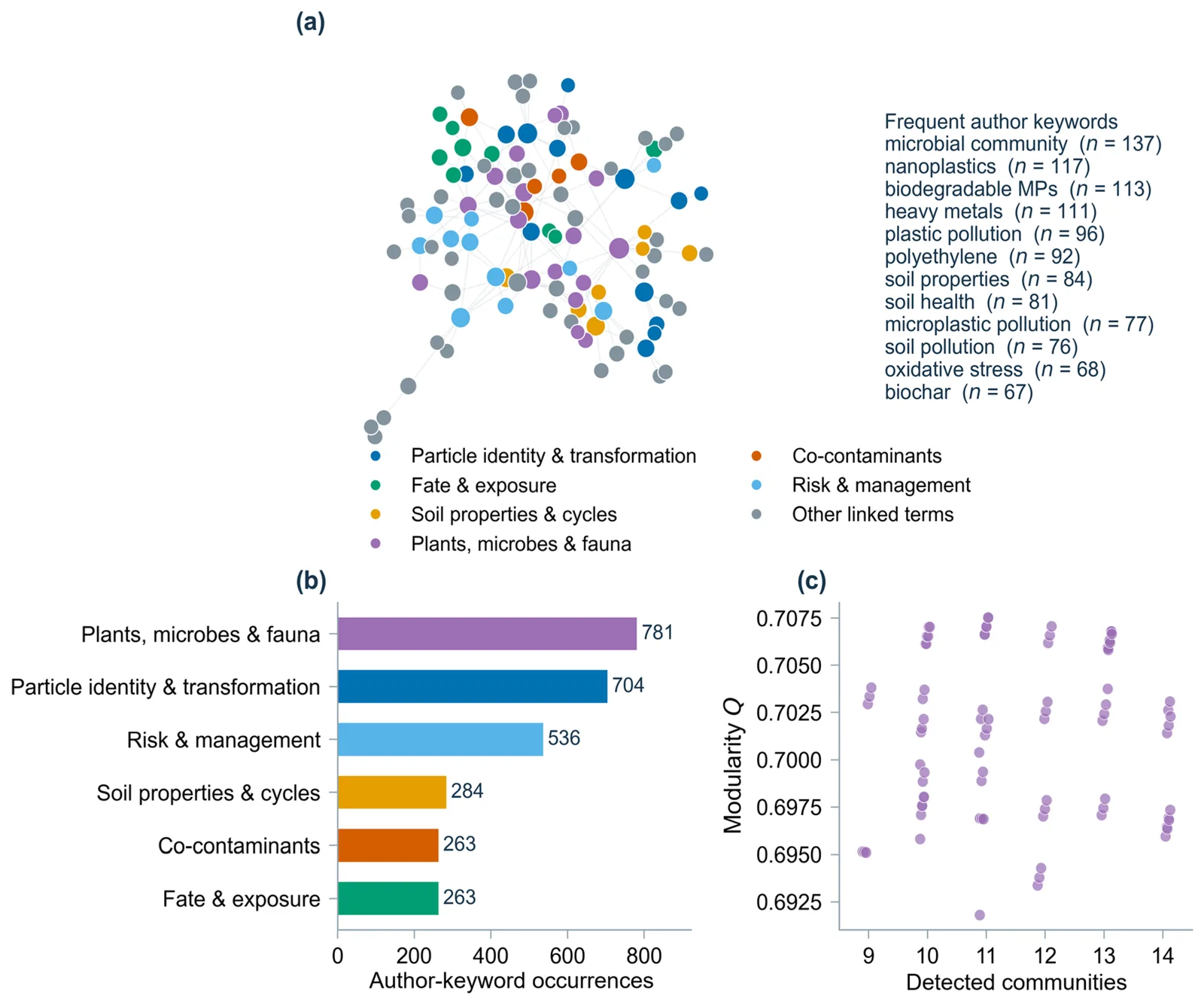
Author-keyword network (**a**), display-domain frequencies (**b**), and keyword-network partition robustness (**c**). Colors in (**a**) indicate display domains; gray marks unmatched terms (45/107 nodes), excluded from (**b**). Purple points in (**c**) represent 90 parameter–seed combinations. *n*, records containing a keyword; *Q*, modularity; MPs, microplastics.

**Figure 6:**
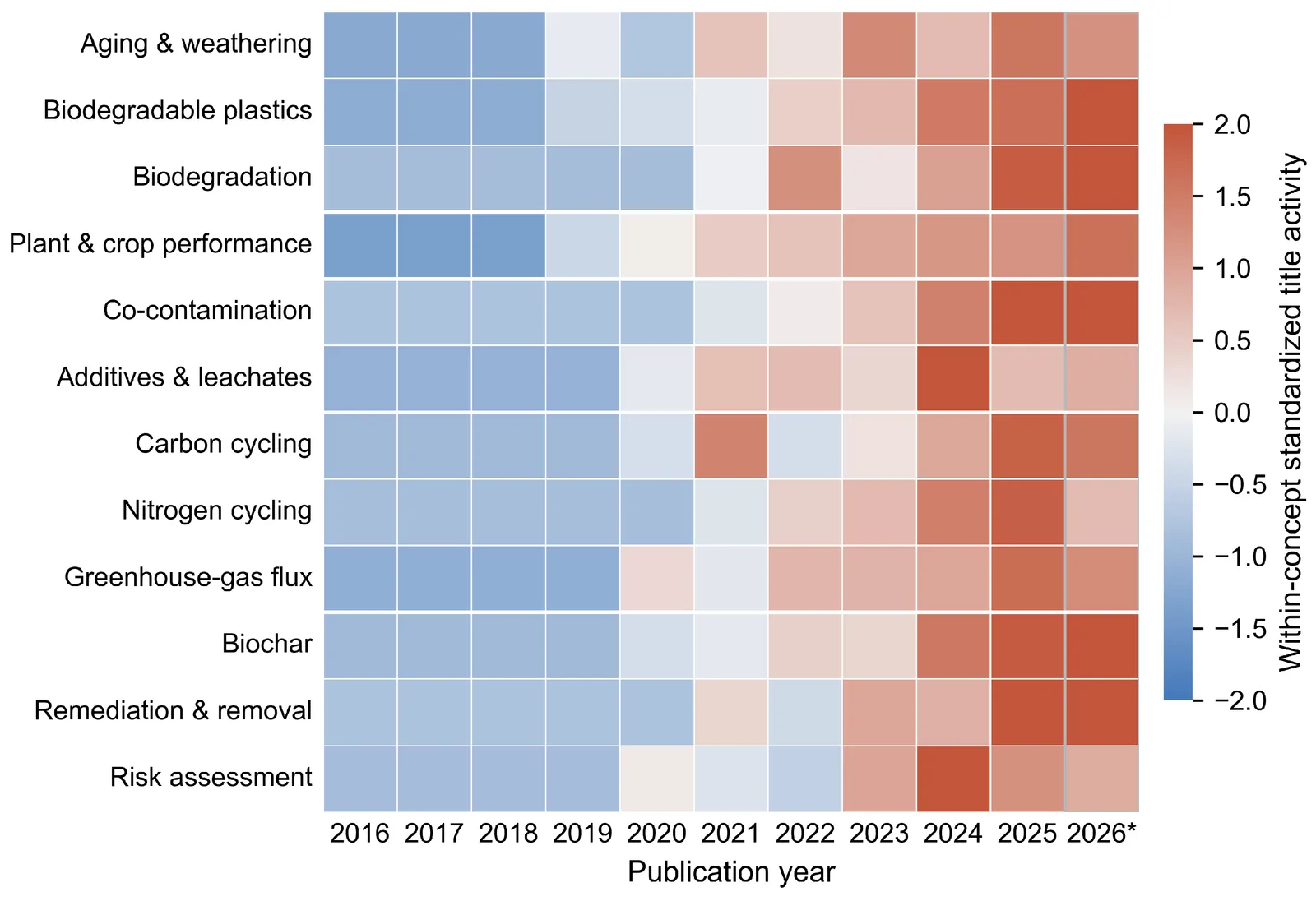
Within-concept standardized annual activity of twelve directionally stable title concepts, 2016–2026. Colors are within-concept z scores relative to 2016–2025, using the population standard deviation and clipped at ±2. The asterisk and gray outline mark incomplete 2026, excluded from trend tests.

**Figure 7:**
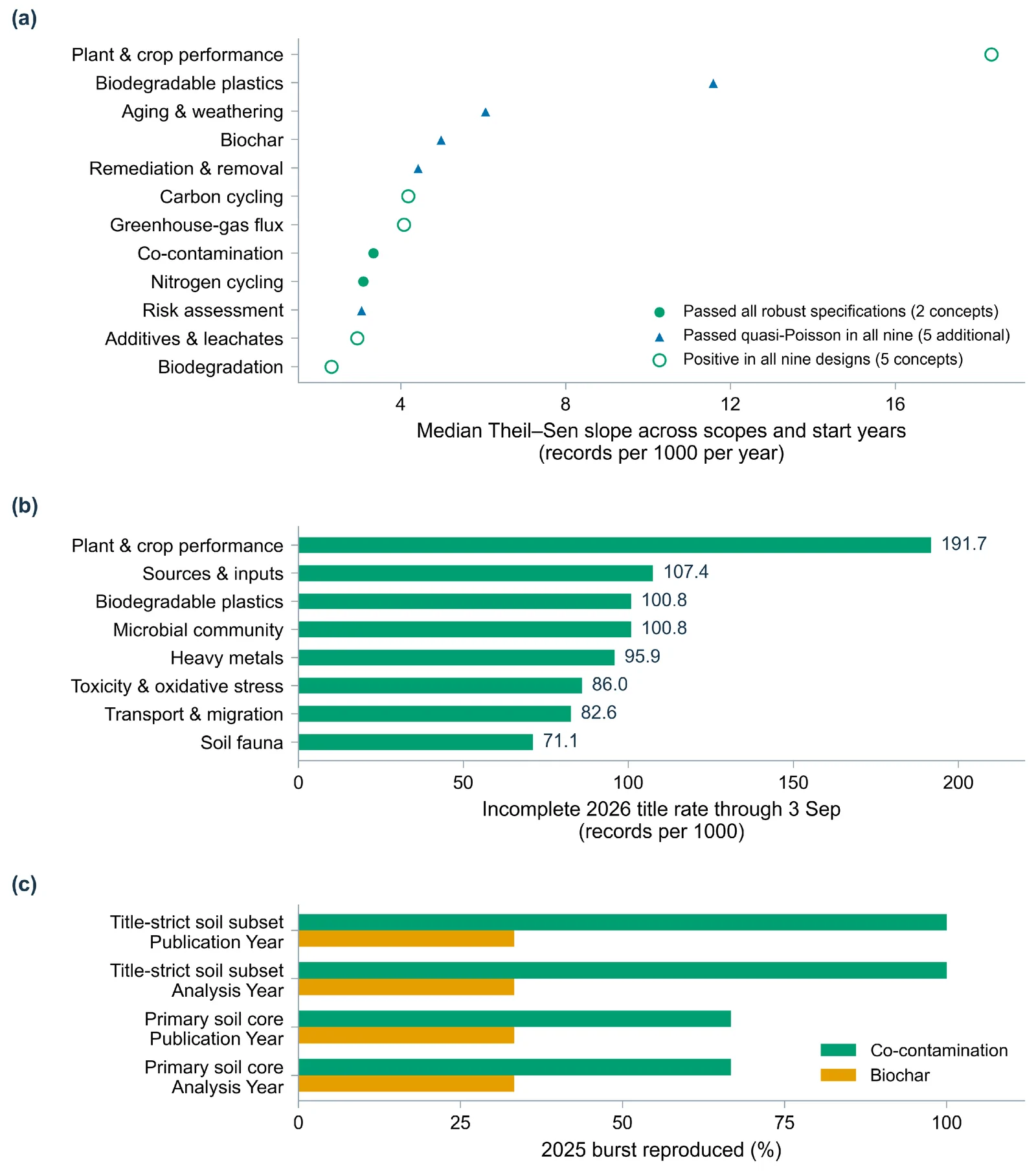
Complete-year trend robustness (**a**), the incomplete-2026 title-concept profile (**b**), and exploratory 2025 burst reproducibility (**c**). In (**a**), green circles pass all robust specifications, blue triangles mark additional concepts passing all quasi-Poisson designs, and open circles retain positive directions. In (**c**), green denotes co-contamination and orange denotes biochar.

**Figure 8:**
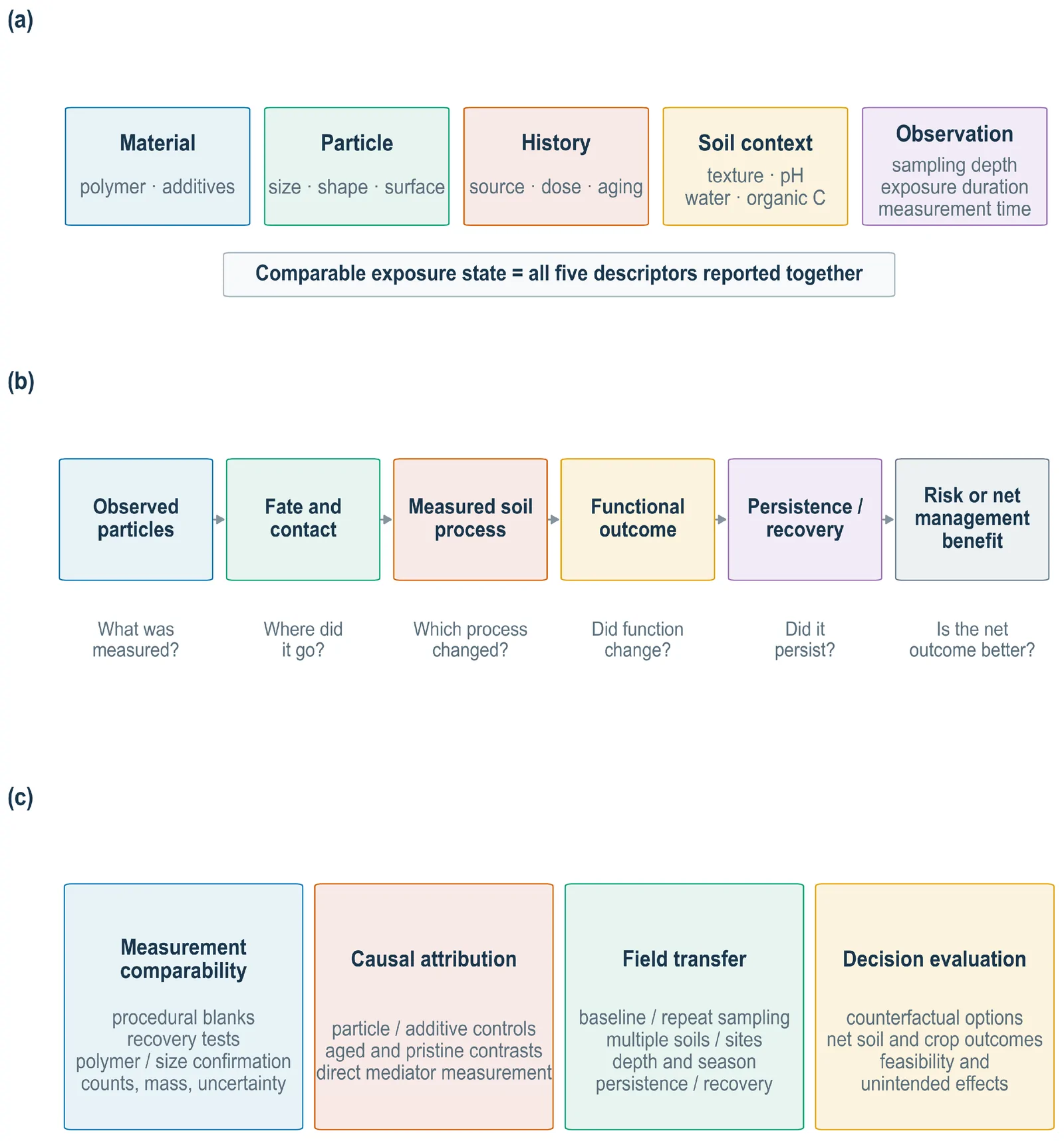
Exposure-state descriptor groups (**a**), the claim-to-evidence chain (**b**), and minimum design requirements (**c**). C, carbon.

**Table 1:** Datasets used in the review, analyses supported, and interpretation limits. WoSCC, Web of Science Core Collection.

Dataset	Records and Period	Analyses Supported	Interpretation Limit
WoSCC retrieval set	5629 raw; 5628 eligible 2013–2026	Export and completeness audit	Recall-oriented retrieval; not the inference corpus
Primary soil core	3200; 2013–2026 search window (first primary record: 2016)	All primary bibliometric analyses	Focus-anchored; 2026 incomplete
Scope sensitivities	Broad: 4787; title-strict: 2375	Annual and title-trend robustness	Tests dependence on retrieval precision
Mechanistic synthesis	Soil experiments, field studies, reviews	Exposure, process, function, management	Conditional synthesis; no pooled effect size
Manual relevance audit	120 sampled primary-corpus records	Independent human check of corpus relevance	Retained-record relevance check; not a recall or inter-reviewer estimate
Article-only sensitivity	2756 articles/article early-access records	Key title trends and co-citation sensitivity	Robustness subset; does not replace mixed-document primary corpus

**Table 2:** Evidence required for progressively stronger conclusions about soil microplastics.

Claim Level	Insufficient Basis	Evidence Required
Comparable exposure	Reported abundance alone	Recovery, blanks, size limits, polymer confirmation, counts and mass, and uncertainty
Causal mechanism	Biological association alone	Factorial or longitudinal separation, temporal order, and direct process endpoints
Persistent field effect	Controlled treatment alone	Baselines, repeated depth profiles, mass balance, multiple soils or sites, and management history
Net management benefit	One reduced hazard alone	Life-cycle comparison, soil function, feasibility, unintended effects, and uncertainty-bounded risk

## Data Availability

The derived data, analysis code, figure-source tables, evidence matrix, and proofreading correction audit are provided in the Supplementary Materials. Raw Web of Science records are subject to Clarivate licensing and are not redistributed in the public archive. Further inquiries can be directed to the corresponding authors.

## Supplementary Materials

The following supporting information can be downloaded at: https://www.mdpi.com/article/10.3390/polym18192364/s1. Supplementary Tables.xlsx: Tables S0–S11 and sub-tables covering corpus screening, bibliographic coverage, title-concept analyses, network robustness, mechanistic evidence, and manual validation. Analytical Audit Workbook.xlsx: analytical outputs, source-verification records, and sensitivity checks. Supplementary_Data_and_Code: derived data, analysis and plotting code, figure-source tables, citation-link checks, and inclusion–exclusion sensitivity (Table S6c). Raw Web of Science records are subject to Clarivate licensing and are not redistributed.
