# Supplementary material for: Exposure States, Environmental Fate, and Functional Effects of Microplastics in Soil: Bibliometric Mapping and Critical Mechanistic Synthesis
Source: Polymers (Basel). 2026 Sep 28;18(19):2364. doi: 10.3390/polym18192364 (PMC13667040; doi:10.3390/polym18192364)
Supplement: Supplementary file 1 [file polymers-18-02364-s001.zip › Analysis/Cocitation/publisher_references_10.1007_s11356-021-18034-9.html]

Microplastic effects on soil system parameters: a meta-analysis study | Environmental Science and Pollution Research | Springer Nature Link


Skip to main content


Advertisement


Log in

Menu

Find a journal

Publish with us

Track your research

Search

Saved research

Cart

1. Home
2. Environmental Science and Pollution Research
3. Article

# Microplastic effects on soil system parameters: a meta-analysis study

- Review Article
- Published: 11 January 2022

- Volume 29, pages 11027–11038 (2022)
- Cite this article

Save article

View saved research

Environmental Science and Pollution Research

Aims and scope

Submit manuscript

- Haixiao Li^1^,
- Le Liu^2^,
- Yan Xu^3^ &
- …
- Junyang Zhang^4^

Show authors

- 6057 Accesses
- 69 Citations
- Explore all metrics

## Abstract

Microplastics are generally considered as an emerging contaminant in the environment due to their toxic additives and transport of other contaminants. However, the potential threats of microplastics in soil should be concerned due to inconsistent research results. In this study, a meta-analysis based on 32 recent relevant studies was conducted to compare the response of soil system parameters including microbial community, aggregate structure, soil nutrient contents, and crop growth to the presence of microplastics. The results showed that microplastics presented no significant effects on soil dissolved organic carbon contents and the amounts of available phosphate, nitrate, and ammonium. Although microplastics would not significantly influence the diversity of soil microorganisms, they could significantly increase soil microorganism amounts with a standard mean difference at 19.32. We also found that microplastics tended to significantly decrease soil water stable macro-aggregate (> 0.25 mm) contents with a significantly negative standard mean difference (− 0.90) in meta-analysis. Moreover, soil microplastics seemed not to affect crop growth by having non-significant effects on both crop under-ground and above-ground biomasses. These results indicate that up to date, the main negative impacts caused by microplastics on soil systems could be their negative functions on soil aggregation.

This is a preview of subscription content, log in via an institution


 to check access.

## Access this article

Log in via an institution

## Subscribe and save

Springer+

from €37.37 /Month

- Starting from 10 chapters or articles per month
- Access and download chapters and articles from more than 300k books and 2,500 journals
- Cancel anytime

View plans

## Buy Now

Buy article PDF 39,95 €

Price includes VAT (China (P.R.))

Instant access to the full article PDF.

Institutional subscriptions

**Fig. 1**

**Fig. 2**

### Similar content being viewed by others

### Soil Aggregates and Organic Carbon Transformation in Response to Microplastics Pollution

Article
02 June 2025

### Invisible threats in soil: Microplastic pollution and its effects on soil health and plant growth

Article
09 April 2025

### Unravelling the emerging threats of microplastics to agroecosystems

Article
26 May 2022

### Explore related subjects

Discover the latest articles, books and news in related subjects, suggested using machine learning.

- Soil and Water Protection
- Soil Physics
- Soil Microbiology
- Soil and Rock Mechanics
- Soil Science
- Soil Chemistry
- Microplastic Pollution in Aquatic Ecosystems

## Availability of data and materials

All data generated or analyzed in this study are included in the published articles mentioned in Table 1.

## References

- Bejgarn S, MacLeod M, Bogdal C, Breitholtz M (2015) Toxicity of leachate from weathering plastics: an exploratory screening study with Nitocra spinipes. Chemosphere 132:114–119

  Article 
  CAS 
  Google Scholar
- Bradl HB (2004) Adsorption of heavy metal ions on soils and soils constituents. J Colloid Interface Sci 277:1–18

  Article 
  CAS 
  Google Scholar
- Cao D, Wang X, Luo X et al (2017) Effects of polystyrene microplastics on the fitness of earthworms in an agricultural soil. IOP Conf Ser: Earth Environ Sci 61:012148

  Article 
  Google Scholar
- Chai B, Li X, Liu H et al (2020a) Bacterial communities on soil microplastic at Guiyu, an E-Waste dismantling zone of China. Ecotoxicol Environ Saf 195:110521
- Chai B, Wei Q, She Y et al (2020b) Soil microplastic pollution in an e-waste dismantling zone of China. Waste Manag 118:291–301

  Article 
  CAS 
  Google Scholar
- Chamas A, Moon H, Zheng J et al (2020) Degradation rates of plastics in the environment. ACS Sustain Chem Eng 8:3494–3511

  Article 
  CAS 
  Google Scholar
- CherifLahimer M, Ayed N, Horriche J, Belgaied S (2017) Characterization of plastic packaging additives: food contact, stability and toxicity. Arab J Chem 10:S1938–S1954

  Article 
  CAS 
  Google Scholar
- Chia RW, Lee J-Y, Kim H, Jang J (2021) Microplastic pollution in soil and groundwater: a review. Environ Chem Lett 19:4211–4224

  Article 
  Google Scholar
- Chu G, Zhao J, Liu Y et al (2019) The relative importance of different carbon structures in biochars to carbamazepine and bisphenol A sorption. J Hazard Mater 373:106–114

  Article 
  CAS 
  Google Scholar
- de Souza Machado AA, Horton AA, Davis T, Maaß S (2020) Microplastics and their effects on soil function as a life-supporting system. In: He D, Luo Y (eds) Microplastics in terrestrial environments: emerging contaminants and major challenges. Springer International Publishing, Cham, pp 199–222

  Chapter 
  Google Scholar
- de Souza Machado AA, Lau CW, Kloas W et al (2019) Microplastics can change soil properties and affect plant performance. Environ Sci Technol 53:6044–6052

  Article 
  Google Scholar
- Deeks JJ, Higgins JP (2007) Statistical algorithms in Review Manager 5
- Dong Y, Gao M, Qiu W, Song Z (2021) Effect of microplastics and arsenic on nutrients and microorganisms in rice rhizosphere soil. Ecotoxicol Environ Saf 211:111899

  Article 
  CAS 
  Google Scholar
- Fei Y, Huang S, Zhang H et al (2020) Response of soil enzyme activities and bacterial communities to the accumulation of microplastics in an acid cropped soil. Sci Total Environ 707:135634

  Article 
  CAS 
  Google Scholar
- Ferreira GVB, Barletta M, Lima ARA et al (2018) High intake rates of microplastics in a Western Atlantic predatory fish, and insights of a direct fishery effect. Environ Pollut 236:706–717

  Article 
  CAS 
  Google Scholar
- Fu S-F, Ding J-N, Zhang Y et al (2018) Exposure to polystyrene nanoplastic leads to inhibition of anaerobic digestion system. Sci Total Environ 625:64–70

  Article 
  CAS 
  Google Scholar
- Furukawa TA, Barbui C, Cipriani A et al (2006) Imputing missing standard deviations in meta-analyses can provide accurate results. J Clin Epidemiol 59:7–10

  Article 
  Google Scholar
- Gao B, Yao H, Li Y, Zhu Y (2020) Microplastic addition alters the microbial community structure and stimulates soil carbon dioxide emissions in vegetable-growing soil. Environ Toxicol Chem 40:352–365

  Article 
  Google Scholar
- Guo J-J, Huang X-P, Xiang L et al (2020) Source, migration and toxicology of microplastics in soil. Environ Int 137:105263

  Article 
  CAS 
  Google Scholar
- Hahladakis JN, Velis CA, Weber R et al (2018) An overview of chemical additives present in plastics: migration, release, fate and environmental impact during their use, disposal and recycling. J Hazard Mater 344:179–199

  Article 
  CAS 
  Google Scholar
- Huang Y, Zhao Y, Wang J et al (2019) LDPE microplastic films alter microbial community composition and enzymatic activities in soil. Environ Pollut 254:112983

  Article 
  CAS 
  Google Scholar
- Huang B, Sun L, Liu M et al (2021) Abundance and distribution characteristics of microplastic in plateau cultivated land of Yunnan Province, China. Environ Sci Pollut Res 28:1675–1688

  Article 
  CAS 
  Google Scholar
- Huerta Lwanga E, Mendoza Vega J, Ku Quej V et al (2017) Field evidence for transfer of plastic debris along a terrestrial food chain. Sci Rep 7:1–7

  Article 
  CAS 
  Google Scholar
- Kim SW, Waldman WR, Rillig MC (2020) Effects of different microplastics on nematodes in the soil environment: tracking the extractable additives using an ecotoxicological approach. bioRxiv 2020.07.07.192278
- Koelmans AA, Besseling E, Shim WJ (2015) Nanoplastics in the aquatic environment. Critical Review. In: Bergmann M, Gutow L, Klages M (eds) Marine anthropogenic litter. Springer International Publishing, Cham, pp 325–340

  Chapter 
  Google Scholar
- Kuzyakov Y, Bogomolova I, Glaser B (2014) Biochar stability in soil: decomposition during eight years and transformation as assessed by compound-specific 14C analysis. Soil Biol Biochem 70:229–236

  Article 
  CAS 
  Google Scholar
- Lehmann A, Rillig MC (2015) Understanding mechanisms of soil biota involvement in soil aggregation: a way forward with saprobic fungi? Soil Biol Biochem 88:298–302

  Article 
  CAS 
  Google Scholar
- Lehmann A, Fitschen K, Rillig M (2019) Abiotic and biotic factors influencing the effect of microplastic on soil aggregation. Soil Syst 3:21

  Article 
  CAS 
  Google Scholar
- Lehmann A, Leifheit EF, Gerdawischke M, Rillig MC (2020) Microplastics have shape- and polymer-dependent effects on soil processes. bioRxiv 2020.06.02.130054
- Lehmann A, Leifheit EF, Gerdawischke M, Rillig MC (2021) Microplastics have shape- and polymer-dependent effects on soil aggregation and organic matter loss – an experimental and meta-analytical approach. Microplast Nanoplast 1:7

  Article 
  Google Scholar
- Leifheit EF, Lehmann A, Rillig MC (2021) Potential effects of microplastic on arbuscular mycorrhizal fungi. Front Plant Sci 12
- Li H, Liu L (2021) Short-term effects of polyethene and polypropylene microplastics on soil phosphorus and nitrogen availability. Chemosphere 132984
- Li H, Li Y, Xu Y, Lu X (2020a) Biochar phosphorus fertilizer effects on soil phosphorus availability. Chemosphere 244:125471
- Li J, Yu G, Pan L et al (2020b) Ciprofloxacin adsorption by biochar derived from co-pyrolysis of sewage sludge and bamboo waste. Environ Sci Pollut Res 27:22806–22817

  Article 
  CAS 
  Google Scholar
- Li H-Z, Zhu D, Lindhardt JH et al (2021) Long-term fertilization history alters effects of microplastics on soil properties, microbial communities, and functions in diverse farmland ecosystem. Environ Sci Technol 55:4658–4668

  Article 
  CAS 
  Google Scholar
- Lian J, Wu J, Xiong H et al (2020a) Impact of polystyrene nanoplastics (PSNPs) on seed germination and seedling growth of wheat (Triticum aestivum L.). J Hazard Mater 385:121620

  Article 
  CAS 
  Google Scholar
- Lian J, Wu J, Zeb A et al (2020b) Do polystyrene nanoplastics affect the toxicity of cadmium to wheat (Triticum aestivum L.)? Environ Pollut 263:114498

  Article 
  CAS 
  Google Scholar
- Lian J, Liu W, Meng L et al (2021) Foliar-applied polystyrene nanoplastics (PSNPs) reduce the growth and nutritional quality of lettuce (Lactuca sativa L.). Environ Pollut 280:116978
- Liang Y, Lehmann A, Ballhausen M-B, et al (2019) Increasing temperature and microplastic fibers jointly influence soil aggregation by saprobic fungi. Front Microbiol 10
- Liu H, Yang X, Liu G et al (2017) Response of soil dissolved organic matter to microplastic addition in Chinese loess soil. Chemosphere 185:907–917

  Article 
  CAS 
  Google Scholar
- Liu M, Lu S, Song Y et al (2018) Microplastic and mesoplastic pollution in farmland soils in suburbs of Shanghai, China. Environ Pollut 242:855–862

  Article 
  CAS 
  Google Scholar
- Liu H, Yang X, Liang C et al (2019) Interactive effects of microplastics and glyphosate on the dynamics of soil dissolved organic matter in a Chinese loess soil. CATENA 182:104177

  Article 
  CAS 
  Google Scholar
- Lozano YM, Aguilar-Trigueros CA, Onandia G, et al (2020a) Effects of microplastics and drought on ecosystem functions and multifunctionality. bioRxiv 2020.07.26.221929
- Lozano YM, Lehnert T, Linck LT, et al (2020b) Microplastic shape, concentration and polymer type affect soil properties and plant biomass. bioRxiv 2020.07.27.223768
- Luo H, Xiang Y, He D et al (2019) Leaching behavior of fluorescent additives from microplastics and the toxicity of leachate to Chlorella vulgaris. Sci Total Environ 678:1–9

  Article 
  CAS 
  Google Scholar
- Meiirkhanuly Z, Koziel JA, Bialowiec A, et al (2020) The proof-of-the concept of biochar floating cover influence on swine manure pH: implications for mitigation of gaseous emissions from area sources. Front Chem 8
- Miao L, Wang P, Hou J et al (2019) Distinct community structure and microbial functions of biofilms colonizing microplastics. Sci Total Environ 650:2395–2402

  Article 
  CAS 
  Google Scholar
- Nizzetto L, Futter M, Langaas S (2016) Are agricultural soils dumps for microplastics of urban origin? Environ Sci Technol 50:10777–10779

  Article 
  CAS 
  Google Scholar
- Qin L, Zhou Z, Dai J et al (2016) Novel N-doped hierarchically porous carbons derived from sustainable shrimp shell for high-performance removal of sulfamethazine and chloramphenicol. J Taiwan Inst Chem Eng 62:228–238

  Article 
  CAS 
  Google Scholar
- Rashid MI, Mujawar LH, Shahzad T et al (2016) Bacteria and fungi can contribute to nutrients bioavailability and aggregate formation in degraded soils. Microbiol Res 183:26–41

  Article 
  CAS 
  Google Scholar
- Rillig MC (2018) Microplastic disguising as soil carbon storage. Environ Sci Technol 52:6079–6080

  Article 
  CAS 
  Google Scholar
- Rillig MC, Ingraffia R, de Souza Machado AA (2017) Microplastic incorporation into soil in agroecosystems. Front Plant Sci 8:1805

  Article 
  Google Scholar
- Rillig MC, Lehmann A, Ryo M, Bergmann J (2019) Shaping up: toward considering the shape and form of pollutants. Environ Sci Technol 53:7925–7926

  Article 
  CAS 
  Google Scholar
- Schöpfer L, Menzel R, Schnepf U, et al (2020) Microplastics effects on reproduction and body length of the soil-dwelling nematode Caenorhabditis elegans. Front Environ Sci 0
- Tang J, Mo Y, Zhang J, Zhang R (2011) Influence of biological aggregating agents associated with microbial population on soil aggregate stability. Appl Soil Ecol 47:153–159

  Article 
  Google Scholar
- Tu C, Zhou Q, Zhang C et al (2020) Biofilms of Microplastics. In: He D, Luo Y (eds) Microplastics in terrestrial environments: emerging contaminants and major challenges. Springer International Publishing, Cham, pp 299–317

  Chapter 
  Google Scholar
- Ügdüler S, Van Geem KM, Roosen M et al (2020) Challenges and opportunities of solvent-based additive extraction methods for plastic recycling. Waste Manag 104:148–182

  Article 
  Google Scholar
- Vogelmann ES, Reichert JM, Prevedello J et al (2013) Can occurrence of soil hydrophobicity promote the increase of aggregates stability? CATENA 110:24–31

  Article 
  CAS 
  Google Scholar
- Wang J, Liu X, Li Y et al (2019) Microplastics as contaminants in the soil environment: a mini-review. Sci Total Environ 691:848–857

  Article 
  CAS 
  Google Scholar
- Wang Y, Wang X, Li Y et al (2020) Biofilm alters tetracycline and copper adsorption behaviors onto polyethylene microplastics. Chem Eng J 392:123808

  Article 
  CAS 
  Google Scholar
- Xu Y, Yu X, Xu B et al (2021) Sorption of pharmaceuticals and personal care products on soil and soil components: influencing factors and mechanisms. Sci Total Environ 753:141891

  Article 
  CAS 
  Google Scholar
- Yan Y, Chen Z, Zhu F, et al (2020) Effect of polyvinyl chloride microplastics on bacterial community and nutrient status in two agricultural soils. Bull Environ Contam Toxicol 1–8
- Yang Y, Liu W, Zhang Z et al (2020) Microplastics provide new microbial niches in aquatic environments. Appl Microbiol Biotechnol 104:6501–6511

  Article 
  CAS 
  Google Scholar
- Ye X, Wang P, Wu Y et al (2020) Microplastic acts as a vector for contaminants: the release behavior of dibutyl phthalate from polyvinyl chloride pipe fragments in water phase. Environ Sci Pollut Res 27:42082–42091

  Article 
  CAS 
  Google Scholar
- Yu Y, Liu Y, Wu L (2013) Sorption and degradation of pharmaceuticals and personal care products (PPCPs) in soils. Environ Sci Pollut Res 20:4261–4267

  Article 
  CAS 
  Google Scholar
- Zang H, Zhou J, Marshall MR et al (2020) Microplastics in the agroecosystem: are they an emerging threat to the plant-soil system? Soil Biol Biochem 148:107926

  Article 
  CAS 
  Google Scholar
- Zeng S, Kan E (2020) Chemical activation of forage grass-derived biochar for treatment of aqueous antibiotic sulfamethoxazole. ACS Omega 5:13793–13801

  Article 
  CAS 
  Google Scholar
- Zeng Z, Tan X, Liu Y, et al (2018) Comprehensive adsorption studies of doxycycline and ciprofloxacin antibiotics by biochars prepared at different temperatures. Front Chem 6
- Zhang GS, Liu YF (2018) The distribution of microplastics in soil aggregate fractions in southwestern China. Sci Total Environ 642:12–20

  Article 
  CAS 
  Google Scholar
- Zhang GS, Zhang FX (2020) Variations in aggregate-associated organic carbon and polyester microfibers resulting from polyester microfibers addition in a clayey soil. Environ Pollut 258:113716

  Article 
  CAS 
  Google Scholar
- Zhang GS, Zhang FX, Li XT (2019) Effects of polyester microfibers on soil physical properties: perception from a field and a pot experiment. Sci Total Environ 670:1–7

  Article 
  CAS 
  Google Scholar
- Zhang B, Yang X, Chen L et al (2020a) Microplastics in soils: a review of possible sources, analytical methods and ecological impacts. J Chem Technol Biotechnol 95:2052–2068

  Article 
  CAS 
  Google Scholar
- Zhang S, Han B, Sun Y, Wang F (2020b) Microplastics influence the adsorption and desorption characteristics of Cd in an agricultural soil. J Hazard Mater 388:121775
- Zheng W, Morris EK, Lehmann A, Rillig MC (2016) Interplay of soil water repellency, soil aggregation and organic carbon. A meta-analysis. Geoderma 283:39–47

  Article 
  CAS 
  Google Scholar
- Zhou Y, Wang J, Zou M et al (2020a) Microplastics in soils: a review of methods, occurrence, fate, transport, ecological and environmental risks. Sci Total Environ 748:141368

  Article 
  CAS 
  Google Scholar
- Zhou Z, Wang C, Luo Y (2020b) Meta-analysis of the impacts of global change factors on soil microbial diversity and functionality. Nat Commun 11:3072

  Article 
  CAS 
  Google Scholar
- Zhu F, Zhu C, Wang C, Gu C (2019) Occurrence and ecological impacts of microplastics in soil systems: a review. Bull Environ Contam Toxicol 102:741–749

  Article 
  CAS 
  Google Scholar

Download references

## Funding

This study was supported by the Fundamental Research Funds for the Central Universities, Nankai University (63191330), the Young Elite Scientists Sponsorship Program by Tianjin (TJSQNTJ-2018–06), and the Youth project of National Natural Science Foundation of China (42107013).

## Author information

### Authors and Affiliations

1. School of Environmental Science and Engineering, Hubei Polytechnic University, Hubei, 435003, Huangshi, China

   Haixiao Li
2. College of Environmental Science and Engineering, Nankai University, Tianjin, 300350, China

   Le Liu
3. Department of Soils and Agri-Food Engineering, Laval University, Paul Comtois Bldg., Quebec City, QC, G1K 7P4, Canada

   Yan Xu
4. Xi’an Jiaotong-Liverpool University, Suzhou, 215123, China

   Junyang Zhang

Authors

1. Haixiao Li

   View author publications

   Search author on:PubMed Google Scholar
2. Le Liu

   View author publications

   Search author on:PubMed Google Scholar
3. Yan Xu

   View author publications

   Search author on:PubMed Google Scholar
4. Junyang Zhang

   View author publications

   Search author on:PubMed Google Scholar

### Contributions

HL is responsible for conceptualization, funding acquisition, investigation, supervision, data curation, formal analysis, software, visualization, and writing—original draft of the study; LL is partly in charge of conceptualization, methodology, and validation of the study; YX is also partly responsible for formal analysis, methodology, and validation of the study; JZ is partly responsible for data curation, formal analysis, software, and visualization for each part of the analysis in the study; all the authors have participated in writing—review and editing work of the manuscript.

### Corresponding author

Correspondence to
Haixiao Li.

## Ethics declarations

### Ethics approval and consent to participate

Not applicable.

### Consent for publication

Not applicable.

### Competing interests

The authors declare no competing interests.

## Additional information

Responsible Editor: Kitae Baek

### Publisher's note

Springer Nature remains neutral with regard to jurisdictional claims in published maps and institutional affiliations.

## Supplementary Information

Below is the link to the electronic supplementary material.

### Supplementary file1 (DOCX 205 KB) (download DOCX )

### Supplementary file2 (XLSX 72 KB) (download XLSX )

## Rights and permissions

Reprints and permissions

## About this article

### Cite this article

Li, H., Liu, L., Xu, Y. *et al.* Microplastic effects on soil system parameters: a meta-analysis study.
*Environ Sci Pollut Res* **29**, 11027–11038 (2022). https://doi.org/10.1007/s11356-021-18034-9

Download citation

- Received: 24 August 2021
- Accepted: 06 December 2021
- Published: 11 January 2022
- Version of record: 11 January 2022
- Issue date: February 2022
- DOI: https://doi.org/10.1007/s11356-021-18034-9

### Share this article

Anyone you share the following link with will be able to read this content:

Get shareable link

Sorry, a shareable link is not currently available for this article.

Copy shareable link to clipboard

Provided by the Springer Nature SharedIt content-sharing initiative

### Keywords

- Microbial community
- Soil aggregation
- Soil nutrient availability
- Crop biomass

## Access this article

Log in via an institution

## Subscribe and save

Springer+

from €37.37 /Month

- Starting from 10 chapters or articles per month
- Access and download chapters and articles from more than 300k books and 2,500 journals
- Cancel anytime

View plans

## Buy Now

Buy article PDF 39,95 €

Price includes VAT (China (P.R.))

Instant access to the full article PDF.

Institutional subscriptions

Advertisement

## Search

Search by keyword or author

Search

## Navigation

- Find a journal
- Publish with us
- Track your research


## Footer Navigation

### Discover content

- Journals A-Z
- Books A-Z
- Subjects A-Z

### Publish with us

- Journal finder
- Publish your research
- Language editing
- Open access publishing

### Products and services

- Our products
- Librarians
- Societies
- Partners and advertisers

### Our brands

- Springer
- Nature Portfolio
- BMC
- Palgrave Macmillan
- Apress
- Discover

### Corporate Navigation

- Your privacy choices/Manage cookies
- Your US state privacy rights
- Accessibility statement
- Terms and conditions
- Privacy policy
- Help and support
- Legal notice
- Cancel contracts here

175.22.82.29

Not affiliated


© 2026 Springer Nature
